# Polarization Decoupling Multi‐Port Beam‐Splitting Metasurface for Miniaturized Magneto‐Optical Trap

**DOI:** 10.1002/advs.202506289

**Published:** 2025-07-12

**Authors:** Tian Tian, Chen Qing, Yuxuan Liao, Jiajun Zhu, Yongzhuo Li, Xue Feng, Dengke Zhang, Yidong Huang

**Affiliations:** ^1^ Department of Electronic Engineering Tsinghua University Beijing 100084 China; ^2^ School of Instrumentation and Optoelectronic Engineering Beihang University Beijing 100191 China

**Keywords:** beam splitting, metasurface, miniaturized magneto‐optical trap, polarization decoupling

## Abstract

In regular magneto‐optical trap (MOT) systems, the delivery of six circularly polarized (CP) cooling beams requires complex and bulky optical arrangements including waveplates, mirrors, retroreflectors, etc. To address such technique challenges, a beam delivery system for miniaturized MOT is proposed entirely based on meta‐devices. The key component is a novel polarization decoupling multi‐port beam‐splitting (PD‐MPBS) metasurface that relies on both propagation phase and geometric phase. The fabricated samples exhibit high beam‐splitting power uniformity (within 4.4%) and polarization purities (91.3–93.2%). By leveraging such a beam‐splitting device as well as a reflective beam‐expanding meta‐device, an integrated six‐beam delivery system for miniaturized MOT application is implemented. The experimental results indicate that six expanded beams have been successfully delivered with uniform power (within 9.5%), the desired CP configuration, and a large overlapping volume (76.2 mm^3^). It is believed that a miniaturized MOT with the proposed beam delivery system is very promising for portable application of cold atom technology in precision measurement, atomic clock, quantum simulation, and computing, etc.

## Graphical abstract

A meta‐device‐based six‐beam delivery system is proposed for miniaturized MOT, featuring a novel polarization decoupling multi‐port beam‐splitting (PD‐MPBS) metasurface using propagation and geometric phases. The fabricated PD‐MPBS samples exhibit high beam‐splitting power uniformity (within 4.4%) and polarization purities (91.3–93.2%). The integrated system delivers six expanded beams with uniform power (within 9.5%), desired polarization configuration, and large overlapping volume (76.2 mm^3^).

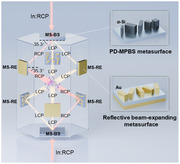

## 1 Introduction

Magneto‐optical trap (MOT), a cornerstone technique for cooling and trapping neutral atoms, was first successfully demonstrated in 1987 by the Raab group at AT&T Bell Laboratory.^[^ ^1^, ^2^ ^]^ A standard 3D MOT system consists of a magnetic trap provided by a pair of anti‐Helmholtz coils and an optical trap formed by three pairs of mutually orthogonal circularly polarized (CP) laser beams, where the magnetic field zero point coinciding with the center of the optical field.^[^ ^3^ ^]^ The cold atomic ensembles obtained through MOT would serve as long‐coherence‐time quantum bits, which are widely applied in the fields of quantum precision measurement,^[^ ^4^, ^5^, ^6^, ^7^ ^]^ quantum sensing,^[^ ^8^, ^9^, ^10^, ^11^ ^]^ and quantum simulation.^[^ ^12^, ^13^, ^14^ ^]^ Specifically, MOT technology facilitates the implementation of atomic optical clocks,^[^ ^15^, ^16^ ^]^ atomic gravimeters,^[^ ^17^, ^18^ ^]^ atomic accelerometers,^[^ ^6^ ^]^ and quantum computing,^[^ ^19^, ^20^ ^]^ establishing it as an indispensable tool in modern atomic physics, geospatial exploration, and various foundational scientific research domains.

However, regular MOT systems are typically constrained by their substantial size and weight. One of the primary limitations stems from the required six cooling laser beams with mutually orthogonal propagation directions and a specific CP configuration that should be introduced into the vacuum chamber.^[^ ^21^ ^]^ Generating such multiple beams usually requires complex optical systems comprising waveplates, mirrors, retroreflectors, lenses, and other components, inevitably leading to a bulky system configuration.^[^ ^22^, ^23^, ^24^ ^]^ Consequently, the development of chip‐based solutions for generating optical fields with desired polarization configurations and propagation directions has emerged as a crucial frontier for MOT‐related researches. In pursuit of miniaturizing beam delivery system, several innovative MOT configurations have been developed, including pyramidal MOT,^[^ ^25^ ^]^ tetrahedral MOT,^[^ ^26^ ^]^ and grating MOT (G‐MOT).^[^ ^27^, ^28^, ^29^, ^30^ ^]^ Although pyramidal MOT has significantly reduced system size, it is required that the downward input beam undergoes two reflections and traverses the atom cloud to generate the vertically upward beam. This configuration renders the axial beam vulnerable to absorption shielding by the atom cloud and scattering at the pyramid's edges. Alternative configurations, such as tetrahedral and grating MOTs based on the four‐beam arrangement, achieve greater compactness due to reduced beam amount. However, these configurations demonstrate compromised performance in terms of atomic trapping capabilities and cooling efficiency when compared to typical six‐beam MOT systems.^[^ ^31^, ^32^, ^33^, ^34^ ^]^

Recently, metasurface‐empowered devices have opened new avenues to miniaturize MOT systems. Metasurface is a 2D array of subwavelength scattering elements, which are referred to as “meta‐atoms.”^[^ ^35^ ^]^ By precisely tailoring the geometric dimensions and spatial orientations of meta‐atoms, metasurfaces exhibit overwhelming superiority over traditional optical elements, including novel functionalities such as flexible wavefront shaping,^[^ ^36^, ^37^, ^38^ ^]^ polarization transformation,^[^ ^39^, ^40^, ^41^, ^42^ ^]^ and frequency selectivity.^[^ ^43^, ^44^ ^]^ These advancements have spurred the development of diverse miniaturized meta‐devices, including high‐performance metalenses,^[^ ^45^, ^46^, ^47^ ^]^ vortex beam generators,^[^ ^48^ ^]^ invisibility cloaks,^[^ ^49^ ^]^ and meta‐holography.^[^ ^50^ ^]^ Leveraging the flexible beam shaping and polarization control capabilities of metasurfaces, one can replace bulky optical components with meta‐devices to delivery six beams with specific CP configuration and directions required in MOT. Based on metasurfaces, several pioneering researches have demonstrated miniaturized six‐beam MOT systems. Among them, PIC‐launched structures possess a more compact system size, but would suffer from low efficiency due to the coupling of the waveguide mode on chip to propagating mode in free space,^[^ ^51^, ^52^, ^53^ ^]^ which is quite fatal for quantum applications. For free‐space illuminated schemes, despite metasurfaces are employed to partially substitute bulky optical components, the assistance of additional mirrors and retroreflectors remains necessary,^[^ ^54^, ^55^ ^]^ thereby limiting the integration of MOT systems. So far, there is a lack of a fully integrated metasurface‐based MOT scheme under free‐space illuminating.

In this paper, we have proposed and demonstrated a six‐beam delivery system for miniaturized MOT. In our work, six large‐diameter beams are delivered entirely through meta‐devices instead of relying on external bulky optical elements. Specifically, a novel polarization decoupling multi‐port beam‐splitting (PD‐MPBS) based on metasurface is implemented to achieve the functionalities of multiple beam splitting and independent polarization manipulation. For the PD‐MPBS metasurface, both propagation phase and geometric phase are employed to decouple the amplitude and phase modulation for orthogonal polarization components. For the fabricated three‐port PD‐MPBS samples, the measured power differences between the splitting sub‐beams are within 4.4%, and the polarization purities of the sub‐beams are 91.3–93.2%. Besides, a reflective beam‐expanding metasurface is also demonstrated to expand the sub‐beams to millimeter‐level diameter. By leveraging the aforementioned two kinds of meta‐devices, a six‐beam delivery system for miniaturized MOT application has been constructed. In the experiment, the generation and overlapping of six expanded beams have been observed. Specifically, the power of each sub‐beam surpasses 1.14 mW, and the power uniformity across the six beams is maintained within 9.5%. The circular polarization purities of six beams range from 88.5% to 90.6%. Additionally, owing to the mode field diameter (MFD) exceeding 5 mm for each beam, the overlapping volume of the six intersecting beams reaches ≈76.2 mm^3^. In summary, the integrated optical system could deliver six beams characterized by uniform power distribution, desired CP configuration, and a large overlapping volume, which are highly promising for MOT systems with enhanced atom capture capacity and ultralow cooling temperature. We anticipate that this metasurface‐endowed miniaturized MOT system could serve as a robust platform for portable cold‐atom technologies in precision measurement, atomic clock, quantum simulation, and quantum computing, etc.

## 2 Results and Discussion

### 2.1 Design Principles

**Figure 1:**
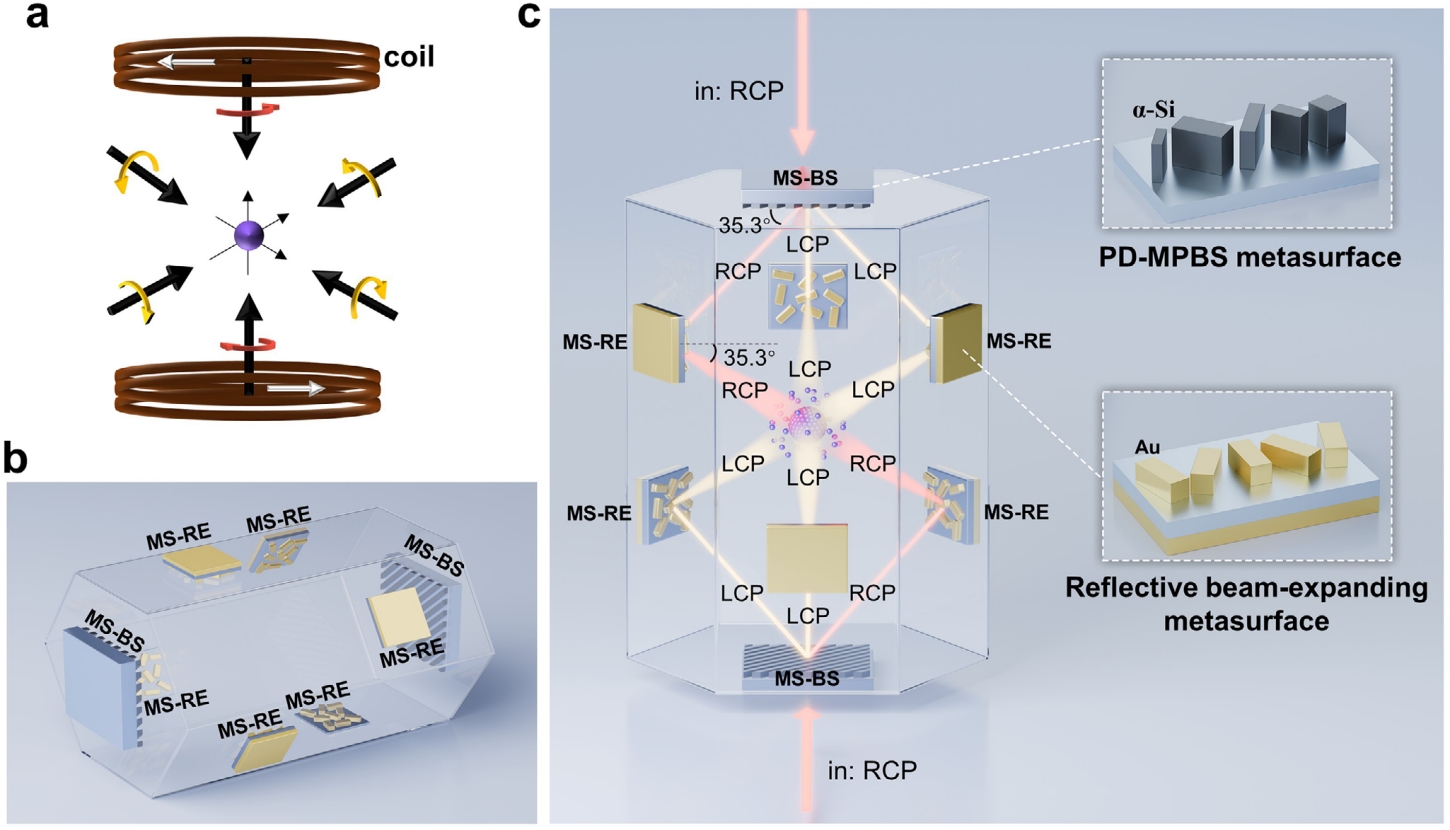
The required configuration of cooling laser beams in a standard 3D MOT and the schematic diagrams of the proposed metasurface‐based six‐beam delivery system for miniaturized MOT. a) The sketch of the required configuration of cooling laser beams in a standard 3D MOT. b) The overall schematic of the proposed metasurface‐based six‐beam delivery system for miniaturized MOT. c) The lateral view of the metasurface‐based beam delivery system and the core meta‐devices.

In a standard 3D MOT system (illustrated in **Figure** **1**a), three pairs of mutually orthogonal CP laser beams with equal optical power are required to suppress the random thermal motion of atoms. Among them, two beams are aligned with the magnetic field coil axis (denoted as axial beams) and supposed to possess opposite helicity relative to the other four radial beams. Moreover, the MFDs of all beams are preferable to be millimeter scale so that enough quantity of atoms can be trapped. To reduce the MOT system size and meet the aforementioned requirements simultaneously, we have proposed a beam delivery system for miniaturized MOT based on meta‐devices, which is schematically shown in Figure 1b. Such a scheme involves two PD‐MPBS metasurfaces (each denoted as MS‐BS) and six reflective beam‐expanding metasurfaces (denoted as MS‐RE). The PD‐MPBS metasurface is engineered to deliver three pairs of counter‐propagating beams with desired propagation directions and polarization configuration, while the reflective beam‐expanding metasurface is designed to enlarge the beam diameter to the millimeter scale. In our scheme, to maintain the orthogonality of three pairs of beams, a hexagonal prism glass chamber is also employed, and all metasurfaces are adhered on the corresponding outside surfaces. The chamber was customized to be filled with rubidium (Rb) atomic vapor and the specific size is provided in Figure S1 (Supporting Information).

The schematic of the whole system from a lateral view is shown in Figure 1c. For the sake of versatility, we have utilized rectangle nanofins as meta‐atoms for both two kinds of meta‐devices (MS‐BS & MS‐RE) in design. Specifically, for the operation wavelength of 780 nm, which corresponds to the D_2_ line transition of Rb atoms, amorphous silicon (α‐Si) and gold (Au) nanofins are adopted for high transmittance and reflectance, respectively. For the PD‐MPBS metasurface, composite phase modulation is employed to achieve three‐port polarization‐decoupling beam splitting, which will be elaborated in the subsequent section. For the reflective metasurface, a pure geometric phase is employed, which only depends on the rotation angles of the anisotropic nanofins (See the inset of MS‐RE in Figure 1c). Since geometric phase only takes effect on the cross‐polarization channels, we elaborately avoid the unwanted co‐polarization component by designing each meta‐atom (Au nanofin) of MS‐RE to introduce a phase difference of *π* between the fast and slow axes (similar to a local half‐wave plate in transmission). As a result, the reflective metasurface acts as a polarization‐preserving device since the rotation direction of the electric field and the direction of the wavevector simultaneously flip upon reflection.

In Figure 1c, the beam trajectories are depicted with two colors representing different CP states. In this work, the mode profiles of all beams are specified as fundamental Gaussian mode since it is commonly utilized in spatial optical systems. From the top side of the hexagonal prism cell, an incident right circular polarization (RCP) Gaussian beam is split by the PD‐MPBS metasurface (MS‐BS) into one RCP beam and two left circular polarization (LCP) beams with uniform power ratio, and then the sub‐beams are directed toward three non‐adjacent sidewalls of the hexagonal prism cell. To achieve mutual orthogonality among the splitting sub‐beams, the splitting angle of the PD‐MPBS metasurface is designed to be 54.7°, corresponding to the included angle of 35.3° with respect to the metasurface plane. Following this, such three sub‐beams are individually reflected and expanded by the corresponding MS‐REs, and finally converge at the center of the cell with expanding MFDs of ≈5 mm. Besides, to generate the other three counter‐propagating beams, an identical optical path is symmetrically arranged at the bottom side of the cell. Finally, the overlapped six sub‐beams would enable cooling and trapping of the released Rb atomic vapor.

As mentioned above, in our scheme, two kinds of meta‐devices are both constructed with anisotropic nanofins. Hence, for generality, the Jones matrix formulation is employed for the device design. For a polarization dependent meta‐atom, when both the propagation phase and geometric phase are considered, the Jones matrix in CP basis is expressed as:^[^ ^56^ ^]^

(1) $$\begin{array}{cc} & J=R_{c}\left(-\theta \right)\left[\begin{array}{c} A_{RR}e^{i\phi_{RR}}\;\;\;\;\;\;A_{RL}e^{i\phi_{RL}}\\ A_{LR}e^{i\phi_{LR}}A_{LL}e^{i\phi_{LL}} \end{array}\right]\\ & R_{c}\left(\theta \right)=\left[\begin{array}{cc} A_{RR}e^{i\phi_{RR}} & A_{RL}e^{i\left(2\theta +\phi_{RL}\right)}\\ A_{LR}e^{i\left(-2\theta +\phi_{LR}\right)} & A_{LL}e^{i\phi_{LL}} \end{array}\right] \end{array}$$

where, θ refers the in‐plane rotation angle of the meta‐atom, and *R*_c_(θ) is the rotation matrix. *A_RR_*,*A_RL_*,*A_LR_*,*A_LL_* denotes the normalized transmitted amplitude, and the first/second letter in subscript (*R*/*L*) refers the CP state of the incident/transmitted light, respectively. Similarly, ϕ_*RR*_,ϕ_*RL*_,ϕ_*LR*_,ϕ_*LL*_ denotes the propagation phase.

Additionally, when the nanofin structure is considered as a meta‐atom, the following relations can be derived based on mirror symmetry: *A_RR_* = *A_LL_*, *A_RL_* = *A_LR_* ,  ϕ _*RR*_ = ϕ_*LL*_, ϕ_*RL*_ = ϕ_*LR*_ . Under these conditions, if the incident light is RCP state, the output electric field *E*_out_ can be written as:

(2) $$\begin{array}{cc} E_{\mathrm{out}} & =J\;E_{\mathrm{in}}=\left[\begin{array}{cc} A_{RR}e^{i\phi_{RR}} & A_{RL}e^{i\left(2\theta +\phi_{RL}\right)}\\ A_{RL}e^{i\left(-2\theta +\phi_{RL}\right)} & A_{RR}e^{i\phi_{RR}} \end{array}\right]\;\left[\begin{array}{c} 1\\ 0 \end{array}\right]\\ & =\left[\begin{array}{c} A_{RR}e^{i\phi_{RR}}\\ A_{RL}e^{i\left(-2\theta +\phi_{RL}\right)} \end{array}\right] \end{array}$$

Further, Equation (2) can be divided into two cases according to the required meta‐devices. First, for the reflective beam‐expanding metasurface, the simplest geometric phase‐only modulation is implemented, indicating that the propagation phases (ϕ_*RR*_ and ϕ_*RL*_) equal to 0. Besides, since the nanofins act as local half‐wave plates (analogous to the transmission mode), Equation 2 can be simplified as $E_{out}^{MS-RE}=\left[\begin{array}{c} \;0\\ e^{-2\theta i} \end{array}\right]\;$. Thus, arbitrary phase distribution can be conveniently obtained by simply altering the rotation angles of nanofins via the geometric phase principle. On the other hand, for the PD‐MPBS metasurface, the geometric phase is incapable of achieving polarization‐decoupling. Here, both the propagation phase and geometric phase are employed to increase the design degrees of freedom. As shown in Equation 2, the phase term of the two output polarization components can be manipulated independently. Specifically, the output co‐polarization component (i.e., RCP) is modulated solely by propagation phase while the cross‐polarization component (LCP) is modulated by both propagation phase and the geometric phase. Additionally, the amplitude coefficient ratio (*A_RR_*/*A_RL_*) can be tailored by selecting the proper meta‐atoms. Thus, through combining propagation and geometric phase, it is possible to modulate both the amplitude and phase of the two polarization components independently.

Based on the above analyses, different strategies of manipulating the phase response are employed for the design of two desired meta‐devices. Subsequently, the specific device structure could be obtained with corresponding strategies. For both meta‐devices, the design process can be summarized as three steps. The first step is to design the corresponding target phase function to implement PD‐MPBS/beam‐expanding functionality. Following this, the next step is establishing a proper “meta‐atom library” which covers the phase modulation range of 0–2*π*. Finally, by referring to the meta‐atom library, the target phase function is mapped to the specific distributions of meta‐atom structural parameters. In followed sections, the details of designing each meta‐device would be elaborated in turn.

**Figure 2:**
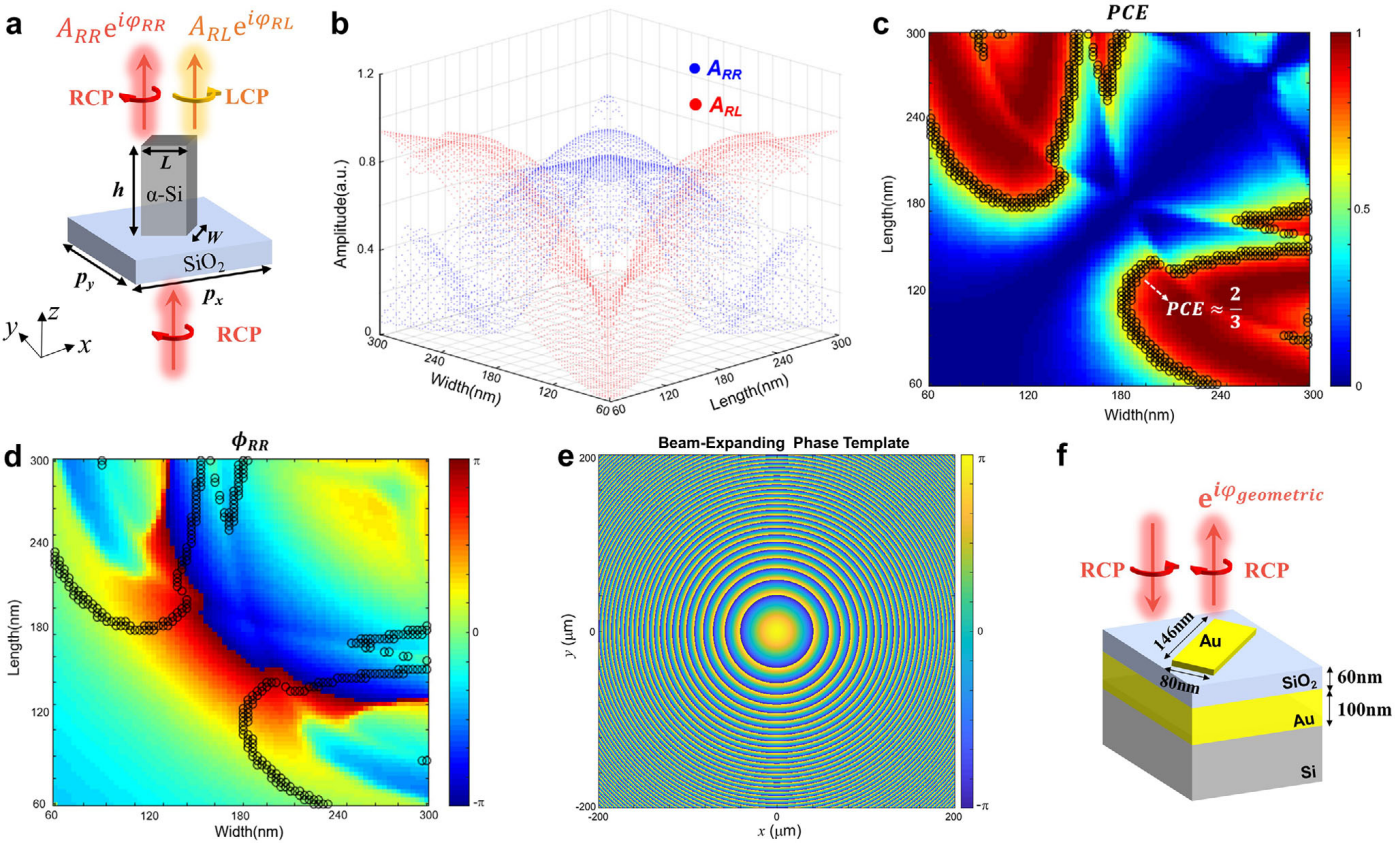
Design principles of the proposed PD‐MPBS and reflective beam‐expanding metasurfaces. a) The schematic of the rectangle α‐Si nanofin. b) The normalized amplitude of transmitted RCP and LCP components for different sizes of α‐Si nanofins. c) *PCE* for different sizes of α‐Si nanofins. d) The phase delay of the transmitted RCP component for different sizes of α‐Si nanofins. e) The beam‐expanding phase template (*f*  =   − 1.1 mm) for reflective beam‐expanding metasurface. f) The schematic of Au‐SiO_2_‐Au nanofin.

First, for the PD‐MPBS metasurface, the target phase functions for co‐ and cross‐polarization components are different. For the co‐polarization (RCP) component, the target phase function (denoted as ϕ_1_) is designed to achieve beam deflecting with a deflection angle of 54.7°, corresponding to an ordinary blazed grating profile. While for the cross‐polarization (LCP) component, the target phase function (denoted as ϕ_2_) is engineered to achieve two‐port beam splitting with both deflection angles of 54.7°. In terms of ϕ_2_, to obtain a beam‐splitting phase pattern with high efficiency and fidelity, an optimization algorithm based on gradient descent is employed. The optimization results are presented in Section S2 (Supporting Information) and a more detailed algorithm principle can be acquired in our previous work.^[^ ^57^ ^]^ Second, an ingenious meta‐atom library is then established through carefully selecting the parameters. The meta‐atom structure is considered as an amorphous silicon (α‐Si) nanofin array on a quartz substrate to construct an all‐dielectric device (**Figure** **2**a). For more accuracy, we have employed spectroscopic ellipsometry to obtain the refractive index and absorption coefficient of α‐Si material (more details are provided in Figure S3, Supporting Information). The height of nanofins is set as 500 nm, and the period is *p_x_* = *p_y_*  = 340 nm. Through finite‐difference time domain (FDTD) simulation, the transmitted amplitude and phase delay are calculated within length (*L*) and a width (*W*) ranging from 60 to 300 nm. Figure 2b reveals that the normalized amplitude coefficients are relevant to the specific nanofin sizes and satisfy the complementary relation of $A_{RR}^{2}+A_{RL}^{2}=1$. Furthermore, the polarization conversion efficiency (*PCE*), which is defined as $PCE=A_{RL}^{2}/\left(A_{RR}^{2}+A_{RL}^{2}\right)$, is calculated and plotted in Figure 2c. To achieve three‐port beam splitting with uniform power ratio, nanofins with *PCE* ≈ 2/3 are carefully selected and marked with black circles in Figure 2c, thereby establishing a meta‐atom library with 143 nanofins to satisfy the target power ratio. Meanwhile, for the selected nanofins, the phase delay ϕ_*RR*_ can nearly cover from 0 to 2*π* (Figure 2d), which allows for arbitrary target phase function mapping.

Following the established meta‐atom library, the target phase function can be independently implemented for both RCP and LCP output components. According to Equation 2, for the RCP component, we discretize the target phase function ϕ_1_ into ψ_1_, which involves 143 phase values. Then, the size distribution (*W* and *L*) of the nanofins can be obtained by setting ψ_1_ = ϕ_*RR*_ . Next, through setting ϕ_2_ =   − 2θ + ϕ_*RL*_ (i.e., θ  =  (ϕ_*RL*_ − ϕ_2_)/2), the rotation angle distribution of nanofins can be further obtained. Since the selected meta‐atoms inherently satisfy that the output power of LCP is twice than that of the RCP component, the designed PD‐MPBS metasurface can split the incident RCP light into one RCP beam and two LCP beams with equal power ratio. Actually, the finally constructed metasurface is comprised of nanofins with 143 discretized sizes and continuously varying rotation angles. Additionally, we have also analyzed the broadband characteristics of the designed meta‐atoms, as detailed in Section S4 (Supporting Information).

Besides the PD‐MPBS metasurface, the other required meta‐device is the reflective beam‐expanding metasurface. Similar to the previous design steps, the first aim is to obtain a beam‐expanding phase function to enlarge the reflected beam to MFD≈5 mm. The typical beam‐expanding phase function is expressed as:

(3) $$\phi_{expanding}=\frac{2\pi }{\lambda }\;\left(\sqrt{x^{2}+y^{2}+f^{2}}-f\right)$$

where, λ is the operation wavelength. *x* and *y* are the position coordinates of a specific point on the transverse plane. *f* (*f* < 0) refers the focal length of the diverging lens.

To achieve beam expansion to MFD≈5 mm after propagating 16.7 mm (corresponding to the size of the customized hexagonal prism, see details in Section S1, Supporting Information), the focal length (*f*) is iteratively optimized and finally determined to be −1.1 mm. The visualized pattern of the beam‐expanding phase template is shown in Figure 2e. Subsequently, the reflective meta‐atom library is also established. The metal‐insulator‐metal (MIM) structure is employed as a meta‐atom for high reflectance (see Figure 2f). The top layer is a 20 nm‐thick Au rectangular nanofin, and the insulator and bottom layer are 60 nm‐thick silicon dioxide (SiO_2_) and 100 nm‐thick Au, respectively. The period is set as *p_x_* = *p_y_*  = 200 nm. Through parameter scanning, the Au nanofin with dimensions of *L* = 146 nm and *W* = 80 nm is picked out, which can impose the phase delay of *π* between the fast and slow axes while maintaining near‐unity reflectivity. After that, the target beam‐expanding phase template can be mapped into the rotation angle distribution of the Au nanofin array, thereby implementing the desired reflective beam‐expanding metasurface.

### 2.2 Characterization Results of the Proposed Metasurfaces

**Figure 3:**
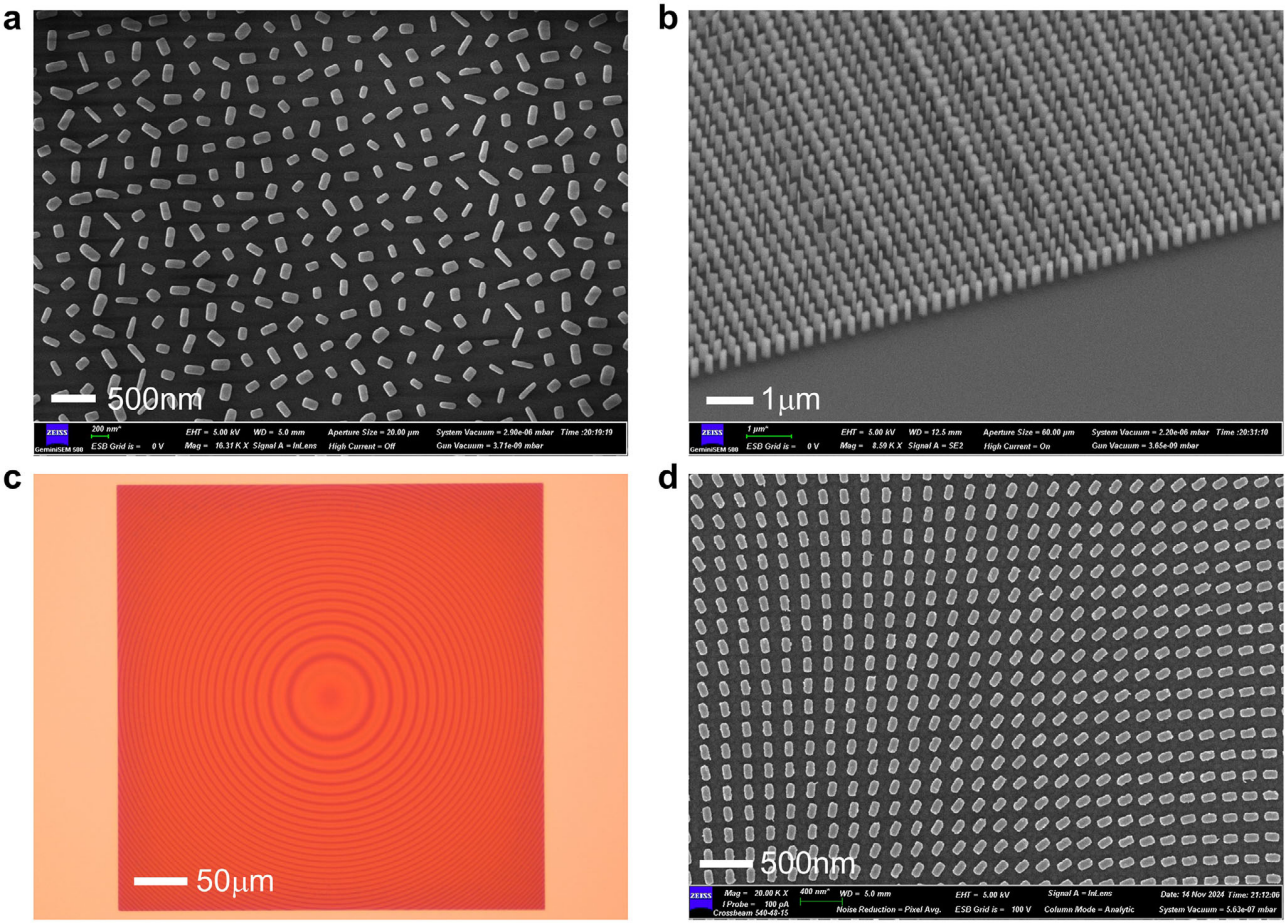
The SEM and optical microscope images of the fabricated PD‐MPBS and reflective beam‐expanding metasurface samples. a) The SEM image of the PD‐MPBS metasurface. b) The SEM image from an oblique viewing angle of the PD‐MPBS metasurface. c) The overall optical microscope image of the reflective beam‐expanding metasurface. d) The partial SEM image of the reflective beam‐expanding metasurface.

To verify our proposal, both the PD‐MPBS and reflective beam‐expanding metasurfaces are fabricated and characterized, respectively. For the fabrication of PD‐MPBS metasurface, a 500 nm‐thick α‐Si layer was first deposited on a quartz substrate via plasma enhanced chemical vapor deposition (PECVD), and then an α‐Si nanofin array was prepared via electron beam lithography (EBL) and inductively coupled plasma reactive ion etching (ICP‐RIE). The detailed preparation process can be found in Section S5.1 (Supporting Information). For the sake of MOT requirements, two samples of PD‐MPBS metasurface were fabricated with the same dimension of 200 µm×200 µm. The scanning electron microscope (SEM) images of one sample are presented in **Figure** **3**a,b. It can be seen that the fabricated α‐Si nanofins have distinct dimensions and orientations. On the other hand, for the MIM‐type beam‐expanding metasurface, the Au and SiO_2_ film was deposited on a crystalline silicon (Si) substrate by electron beam evaporation (EBE) and magnetron sputtering in sequence. During the fabrication process, a 5 nm‐thick chromium (Cr) adhesion layer was incorporated between each Au layer and Si/SiO_2_ dielectric medium to prevent the peeling of the Au layer. After that, Au nanofins were patterned through EBL and lift‐off processes. The diagram of the process flow is provided in Section S5.2 (Supporting Information). Six samples of reflective beam‐expanding metasurface were fabricated with the same dimension of 400 µm×400 µm. Figure 3c,d presents the optical microscope and SEM images of one fabricated sample, respectively. The overall pattern of the sample (Figure 3c) is consistent with the designed phase template (Figure 2e). As shown in Figure 3d, all Au rectangular nanofins possess nearly identical dimensions but varied rotation angles. Additionally, it is worth mentioning that the rectangular edges for both α‐Si and Au nanofins have rounded corners. It is attributed to the shorter dwell time of the electron beam, which usually occurs in the EBL process.

**Figure 4:**
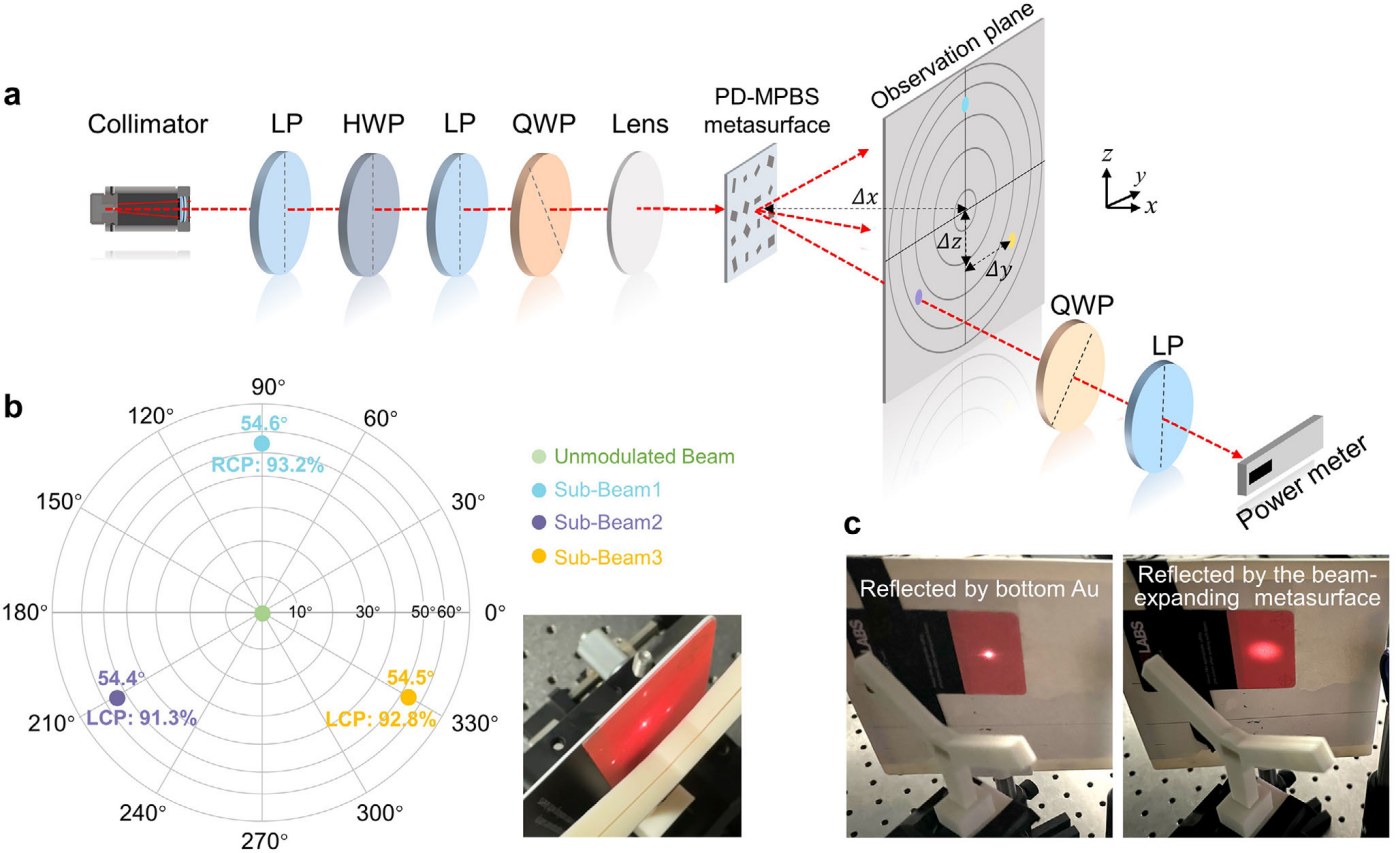
The experimental measurement and characterization results of the fabricated PD‐MPBS and reflective beam‐expanding metasurfaces. a) The schematic of the experimental measurement platform for the PD‐MPBS metasurface. b) The experimental results of the PD‐MPBS metasurface. c) The experimental results of the reflective beam‐expanding metasurface.

To characterize the fabricated PD‐MPBS samples, a testing system has been built up as schematically shown in **Figure** **4**a. The incident laser beam with a wavelength of 780 nm is converted into the RCP state through multiple wave plates, and subsequently focused onto the metasurface by a lens of *f*  = 50 mm. The transmitted sub‐beams are presented on the observation plane. For each sub‐beam, with the recorded distance between the metasurface and observation plane (Δ*x*), as well as the displacements Δ*y* and Δ*z* along the *y*‐axis and *z*‐axis on the observation plane, we can calculate the splitting/deflecting angle θ_*i*_(*i*  = 1,2,3) through:

(4) $$\theta_{i}=\mathrm{ta}n^{-1}\;\left(\frac{\sqrt{\Delta y_{i}^{2}+\Delta z_{i}^{2}}}{\Delta x_{i}}\right)$$

Additionally, the optical power of each sub‐beam is measured by a free‐space optical power meter. In order to evaluate the polarization state of each transmitted sub‐beam, a quarter‐wave plate (QWP) followed by a linear polarizer (LP) are settled along the propagation axis of each sub‐beam, allowing for recording the power of RCP and LCP components independently. Here, we employ the parameter of polarization purity to quantize polarization decoupling efficiency, which is formulated as:

(5) $$Purity\left(RCP\right)=\frac{P_{R}}{P_{R}+P_{L}};\;Purity\left(LCP\right)=\frac{P_{L}}{P_{R}+P_{L}}\;$$

where, *P*_*R*/*L*_ refers the measured optical power of the RCP/LCP component, respectively.

The measured results for the PD‐MPBS metasurface are plotted in Figure 4b. From the original photograph (the inset in the bottom right of Figure 4b), it can be seen that the incident beam is split into three sub‐beams after passing through the metasurface. According to the recorded data, three sub‐beams are located along a circle with an angular separation of ≈120° between adjacent two beams, and the beam‐splitting angles are calculated to be 54.6°, 54.4°, and 54.5° respectively. These results are consistent with the design. It is worth mentioning that the beam‐splitting angle (54.7°) corresponds to the phase gradient of 6.6 × 10^6^ rad m^−1^, which is quite challenging to achieve for conventional optical devices. To our knowledge, such a large phase gradient variation can only be demonstrated with the subwavelength metasurface so far. Additionally, the measured polarization purity for the respective sub‐beam is 93.2%(RCP), 91.3%(LCP), 92.8%(LCP). For both of the fabricated samples, the power deviations between the three sub‐beams are within 4.4% (See complete results of the two samples in Table S1 of Section S6, Supporting Information), indicating high beam‐splitting ratio fidelity for the proposed PD‐MPBS meta‐device. As evident from Figure 4b, a 0‐order central spot (unmodulated beam) is also observed. The presence of a central spot is primarily attributed to fabrication imperfections of nanofins (See the detailed analysis of the impact of fabrication errors in Section S7, Supporting Information), and it is usually observed in metasurface‐related experiments due to the inevitability of fabrication error.^[^ ^54^, ^57^, ^58^ ^]^ The measured diffraction efficiency, defined as the ratio of total intensity at target diffraction orders to the incident intensity, reaches 24.2%. In fact, the theoretical diffraction efficiency is intrinsically limited owing to the large beam‐splitting angle (54.7°), which is detailedly explained in Section S8 (Supporting Information). With improved fabrication technics, it is potential to reduce the proportion of the central spot and thus increase the diffraction efficiency of the metasurface.

On the other hand, the performance of the reflective beam‐expanding metasurface is also measured. As shown in Figure 4c, when the incident Gaussian beam (MFD≈320 µm) is misaligned with the metasurface structure, the light beam reflected by the bottom Au layer of the un‐patterned region on the chip would not exhibit beam expansion. In contrast, when properly aligned with the metasurface structure, the reflected light beam would be significantly expanded. The measurement result indicates that the reflected beam could expand to an MFD of ≈5.1 mm after propagating 16.7 mm, which achieves 4.6‐fold beam expansion compared to directly reflected light. Besides, the polarization‐preserving purity of the output light was measured to be 96.4%, and the average modulation efficiency (i.e., the ratio of reflected power to the incident power) of the fabricated six samples is calculated as high as 88.7%. These experimental results confirm that the reflective beam‐expanding metasurface performs in accordance with the design.

### 2.3 Experimental Setups of Six‐Beam Delivery System for Miniaturized MOT

**Figure 5:**
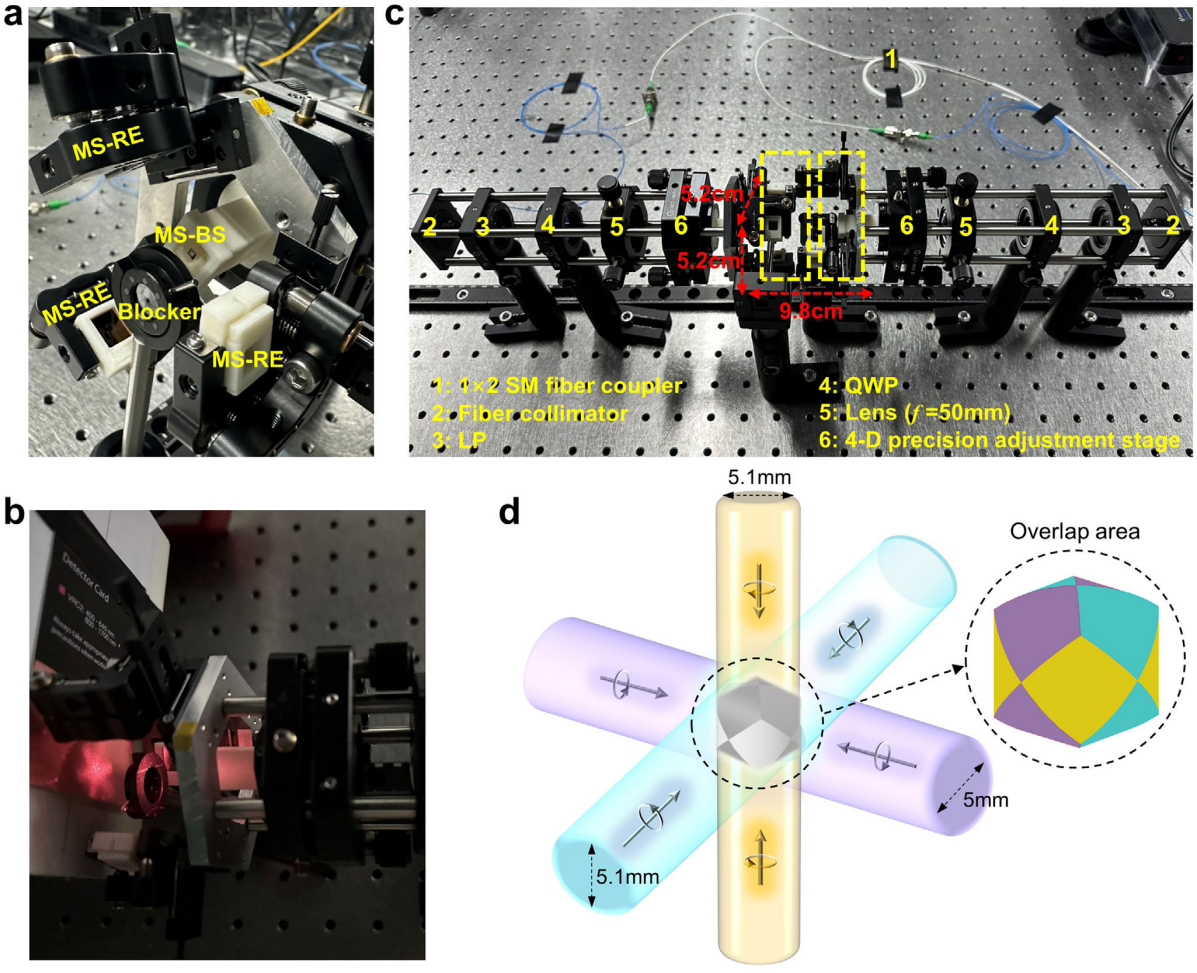
The experimental setup and characterization of a metasurface‐based beam delivery system for miniaturized MOT. a) The lateral photograph of half of the delivery system. b) The overlapping photograph of three expanded beams for half of the delivery system. c) The complete six‐beam delivery system based on metasurfaces for miniaturized MOT. d) The overlapping scenario of six expanded sub‐beams in simulation.

After validating the functionalities of the proposed PD‐MPBS and reflective beam‐expanding metasurfaces, a six‐beam delivery system for miniaturized MOT application could be further assembled. Due to the spatial symmetry of the proposed metasurface‐based MOT system (see Figure 1c), we first implemented half of the optical path to demonstrate the beam property more clearly. A lateral photograph of the half of the system setup is shown in **Figure** **5**a, which consists of one PD‐MPBS metasurface sample (MS‐BS) and three reflective beam‐expanding metasurface samples (MS‐REs). All the metasurfaces are mounted on customized 3D‐printed holders and secured on micro‐adjustable stages for fine alignment. The MS‐RE samples are positioned at non‐adjacent sides of a hexagon base. Additionally, to block the unmodulated 0‐order light spot from MS‐BS, an aperture stop is placed along the axis of the light path. Owing to the sufficiently large splitting angle (54.7°), the aperture would not block the desired splitting sub‐beams. Through meticulous alignment, the incident 780 nm laser beam can be split by MS‐BS, and then reflected and expanded by the corresponding MS‐REs. Finally, three expanded sub‐beams would overlap in the central region along the axial direction (Figure 5b). From Figure 5b, it can be observed that the overlapping region exhibits a “quasi‐hexagonal star” distribution. This phenomenon is due to the fact that the detection plane is tilted at an angle of 54.7° relative to the propagation direction of the beams, resulting in an axial elongation effect of each beam. It is important to note that when the detection plane is perpendicular to the propagation direction of each beam, the expanded beams would maintain the Gaussian mode field distribution, with detailed illustrations provided in Figure S9 (Supporting Information).

Furthermore, by employing the same method to implement the other half of the optical setup, we have built up a complete six‐beam delivery system for miniaturized MOT, as shown in Figure 5c. The system is left‐right symmetric, and the portions enclosed by the yellow dashed line both correspond to Figure 5a. The overall size of the metasurface assembly region is ≈9.8 mm × 5.2 mm × 5.2 mm, which exhibits dramatical footprint reduction compared to the traditional MOT system. In the experiment, the 780 nm laser source is uniformly divided into two paths by a 1 × 2 fiber coupler, which are collimated as free‐space beams from the left and right sides separately. Subsequently, each incident beam is converted to RCP state and focused onto MS‐BS by a series of wave plates and lens. Through the modulation of metasurface assembly, six expanded beams with specific CP configuration would overlap at the center of the system. **Table** **1** summarizes the power, MFDs, and polarization purities of the six sub‐beams. It can be noticed that the power of sub‐beams 1–3 are slightly higher than that of sub‐beams 4–6. This is due to the splitting ratio deviation of the 1 × 2 fiber coupler, which results in unequal incident power for the two sides of the system (16.9 mW vs 16 mW). Nonetheless, the power differences among the six beams are within 9.5%. Besides, the polarization purities of all six beams exceed 88.5%. It is worth mentioning that the polarization purities of sub‐beams in the assembled system is slightly deteriorated with that measured in individual MS‐BS (Figure 4b). It is the result of the superposition of polarization‐preserving efficiency introduced by the MS‐RE. Advancements in fabrication processes could enhance the experimental polarization purities, as analyzed in Section S7 (Supporting Information). Additionally, we also simulated and evaluated the beam overlap volume, which is another key indicator of the MOT system. Based on the measured characteristics of all sub‐beams, the schematic diagram of the overlapping scenario obtained by simulation is illustrated in Figure 5d. Since all the expanded beams are Gaussian modes, the scenario is similar to the intersecting of three mutually perpendicular cylinders, and the overlap region forms a 3D Steinmetz solid (see the inset in Figure 5d). Thus, the beam overlap volume is ≈76.2 mm^3^, which can be approximately estimated by:

(6) $$V=\left(2-\sqrt{2}\right)\;d_{1}d_{2}d_{3}$$

where *d*_1_, *d*_2_, *d*_3_ denotes the MFDs of the three mutually perpendicular sub‐beams delivered from one side of the system.

**Table 1:** The characterization results of the delivered six sub‐beams.

	Power[mW]	MFD[mm]	Polarization Purity
Sub‐beam 1	1.26	5.1	90.0%(RCP)
Sub‐beam 2	1.22	5.0	88.5%(LCP)
Sub‐beam 3	1.20	5.1	90.3%(LCP)
Sub‐beam 4	1.16	5.1	89.7%(RCP)
Sub‐beam 5	1.14	5.0	89.1%(LCP)
Sub‐beam 6	1.14	5.1	90.6%(LCP)

In summary, it can be concluded that the demonstrated system can deliver six beams with nearly uniform power, MFD exceeding 5 mm, the desired CP configuration as well as an overlapping volume of 76.2 mm^3^. For subsequent research, the transparent hexagonal prism vapor chamber of Rb atoms can be integrated with metasurfaces and settled at the system center to demonstrate atom cooling and trapping. The assembly method for the metasurface‐chamber integration and the influence of the chamber wall thickness are detailed in Section S10 (Supporting Information).

## 3 Conclusion

In summary, to achieve compact MOT systems, we have proposed a miniaturized beam delivery scheme based on novel meta‐devices. Specifically, a PD‐MPBS is demonstrated to split a single CP incident beam into multiple RCP and LCP beams with a decoupling power ratio and specific polarization configuration. For the fabricated samples of PD‐MPBS, the measured power differences between the splitting sub‐beams are within 4.4%, and the polarization purities of the sub‐beams are 91.3–93.2%, along with a large splitting angle of 54.7°. It is worth pointing out that, in contrast to previously reported polarization‐controlled metasurface‐based beam splitters,^[^ ^59^, ^60^, ^61^ ^]^ our proposed PD‐MPBS first demonstrates the completely decoupled polarization and spatial ports under single illumination. Additionally, a reflective beam‐expanding metasurface is also achieved to expand the sub‐beams to the desired diameters. With such two kinds of meta‐devices, a fully integrated six‐beam delivery system for miniaturized MOT application is implemented. With the laser input power of 32.9 mW, the power of each sub‐beam exceeds 1.14 mW, and the corresponding emission efficiency is significantly higher than that of previously reported PIC‐launched miniaturized MOT schemes^[^ ^51^, ^52^, ^53^ ^]^ and also competitive with free‐space metasurface‐based MOT schemes.^[^ ^54^, ^55^ ^]^ The power level can be further increased by employing higher‐power lasers. Besides, the power differences among the six beams are within 9.5%, and the polarization purities are 88.5–90.6%. Benefiting from an MFD of over 5 mm for each beam, the overlap volume of the six intersecting beams is ≈76.2 mm^3^. In brief, the demonstrated system is capable to deliver six beams with uniform power, the desired CP configuration, and a large overlapping volume, which are promising to achieve a MOT system with a considerable number of capturing atoms and low cooling temperature. We believe that such a metasurface‐based miniaturized MOT provides an effective solution for portable application of cold atom technology in precision measurement, quantum simulation, and large‐scale quantum computing, etc. Moreover, based on the proposed composite phase design, arbitrary PD‐MPBS meta‐devices can be constructed for various applications. Hence, the proposed PD‐MBPS metasurface is not only applicable to miniaturize the MOT system, but also holds broad potential for other scenarios requiring multi‐port beam splitting and polarization control, such as polarized light detection and ranging (LiDAR) 3D imaging, augmented reality (AR) displays, and laser guidance systems.

## Conflict of Interest

The authors declare no conflict of interest.

## Author Contributions

D.Z., T.T., C.Q. and X.F. conceived the idea. T.T. designed and performed the simulations, experiments, and data analysis. Y.L. (Yuxuan Liao) contributed significantly to the phase pattern optimization algorithm based on gradient descent. J.Z. assisted to characterize the performance of metasurfaces. Y.L. (Yongzhuo Li) provided useful discussions and comments. T.T. and X.F. wrote the paper. D.Z., C.Q. and Y.H. revised the manuscript. The manuscript was written through contributions of all authors. All authors have given approval to the final version of the manuscript.

## Supporting information



Supporting Information

## Data Availability

The data that support the findings of this study are available from the corresponding author upon reasonable request.

## References

[1] E. L. Raab , M. Prentiss , A. Cable , S. Chu , D. E. Pritchard , Phys. Rev. Lett. 1987, 59, 2631. 10035608 10.1103/PhysRevLett.59.2631

[2] C. S. Adams , E. Riis , Prog. Quantum Electron. 1997, 21, 1.

[3] O. N. Prudnikov , A. V. Taichenachev , V. I. Yudin , J. Exp. Theor. Phys. 2015, 120, 587.

[4] Y. Xu , X. Su , Z. Chai , J. Li , Laser Photonics Rev. 2024, 18, 2300355.

[5] L. Zhou , Z. Y. Xiong , W. Yang , B. Tang , W. C. Peng , K. Hao , R. B. Li , M. Liu , J. Wang , M. S. Zhan , Gen. Relativ. Gravit. 2011, 43, 1931.

[6] K. S. Hardman , P. J. Everitt , G. D. McDonald , P. Manju , P. B. Wigley , M. A. Sooriyabandara , C. C. N. Kuhn , J. E. Debs , J. D. Close , N. P. Robins , Phys. Rev. Lett. 2016, 117, 138501. 27715130 10.1103/PhysRevLett.117.138501

[7] P. Shen , K. W. Madison , J. L. Booth , Metrologia 2020, 57, 025015.

[8] G. Chen , Z. Jin , J. Chen , Sensors Actuators Rep. 2023, 5, 100152.

[9] C. Salducci , Y. Bidel , M. Cadoret , S. Darmon , N. Zahzam , A. Bonnin , S. Schwartz , C. Blanchard , A. Bresson , Sci. Adv. 2024, 10, adq4498. 10.1126/sciadv.adq4498 PMC11524193 39475600

[10] F. Li , R. Kodzius , C. P. Gooneratne , I. G. Foulds , J. Kosel , Microchim. Acta 2014, 181, 1743.

[11] R. García , E. Blanco , M. Domínguez , Sens. Actuators, A 2016, 249, 231.

[12] I. Bloch , J. Dalibard , W. Zwerger , Rev. Mod. Phys. 2008, 80, 885.

[13] A. Micheli , G. K. Brennen , P. Zoller , Nat. Phys. 2006, 2, 341.

[14] C. J. E. Straatsma , M. K. Ivory , J. Duggan , J. Ramirez‐Serrano , D. Z. Anderson , E. A. Salim , Opt. Lett. 2015, 40, 3368. 26176471 10.1364/OL.40.003368

[15] O. Kock , W. He , D. Swierad , L. Smith , J. Hughes , K. Bongs , Y. Singh , Sci. Rep. 2016, 6, 37321. 27857214 10.1038/srep37321 PMC5114543

[16] G. W. Hoth , R. Elvin , M. Wright , B. Lewis , A. S. Arnold , P. F. Griffin , E. Riis , in Optical, Opto‐Atomic, and Entanglement‐Enhanced Precision Metrology, Vol. 10934, SPIE, Bellingham, Washington, USA 2019, pp. 250–257

[17] R. Xu , A. Li , D. Li , J. Yan , Appl. Sci. 2023, 13, 6076.

[18] K. Weng , B. Wu , J. Lin , Y. Zhou , B. Cheng , Q. Lin , J. Opt. Soc. Am. B 2020, 37, 1637.

[19] D. J. Blumenthal , A. Isichenko , N. Chauhan , Opt. Quantum 2024, 2, 444.

[20] D. DeMille , Phys. Rev. Lett. 2002, 88, 067901. 11863853 10.1103/PhysRevLett.88.067901

[21] J. A. Rushton , M. Aldous , M. D. Himsworth , Rev. Sci. Instrum. 2014, 85, 121501. 25554265 10.1063/1.4904066

[22] J. F. Barry , D. J. McCarron , E. B. Norrgard , M. H. Steinecker , D. DeMille , Nature 2014, 512, 286. 25143111 10.1038/nature13634

[23] H. J. Williams , S. Truppe , M. Hambach , L. Caldwell , N. J. Fitch , E. A. Hinds , B. E. Sauer , M. R. Tarbutt , New J. Phys. 2017, 19, 113035.

[24] X. Yu , J. Mo , T. Lu , T. Y. Tan , T. L. Nicholson , Phys. Rev. A 2022, 105, L061101.

[25] K. I. Lee , J. A. Kim , H. R. Noh , W. Jhe , Opt. Lett. 1996, 21, 1177. 19876291 10.1364/ol.21.001177

[26] M. Vangeleyn , P. F. Griffin , E. Riis , A. S. Arnold , Opt. Express 2009, 17, 13601. 19654767 10.1364/oe.17.013601

[27] C. C. Nshii , M. Vangeleyn , J. P. Cotter , P. F. Griffin , E. A. Hinds , C. N. Ironside , P. See , A. G. Sinclair , E. Riis , A. S. Arnold , Nat. Nanotechnol. 2013, 8, 321. 23563845 10.1038/nnano.2013.47

[28] D. S. Barker , P. K. Elgee , A. Sitaram , E. B. Norrgard , N. N. Klimov , G. K. Campbell , S. Eckel , New J. Phys. 2023, 25, 103046.

[29] D. S. Barker , E. B. Norrgard , N. N. Klimov , J. A. Fedchak , J. Scherschligt , S. Eckel , Phys. Rev. Appl. 2019, 11, 064023. 10.1103/physrevapplied.11.064023 PMC7722475 33299903

[30] J. G. H. Franssen , T. C. H. de Raadt , M. A. W. van Ninhuijs , O. J. Luiten , Phys. Rev. Accel. Beams 2019, 22, 023401.

[31] M. Sahelgozin , Doctoral diss., Gottfried Wilhelm Leibniz Universität (Hannover, Germany), 2019, 10.15488/5055.

[32] W. Ketterle , K. B. Davis , M. A. Joffe , A. Martin , D. E. Pritchard , Phys. Rev. Lett. 1993, 70, 2253. 10053514 10.1103/PhysRevLett.70.2253

[33] L. Chen , C.‐J. Huang , X.‐B. Xu , Y.‐C. Zhang , D.‐Q. Ma , Z.‐T. Lu , Z.‐B. Wang , G.‐J. Chen , J.‐Z. Zhang , H. X. Tang , C.‐H. Dong , W. Liu , G.‐Y. Xiang , G.‐C. Guo , C.‐L. Zou , Phys. Rev. Appl. 2022, 17, 034031.

[34] S. Bondza , C. Lisdat , S. Kroker , T. Leopold , Phys. Rev. Appl. 2022, 17, 044002.

[35] N. Yu , P. Genevet , M. A. Kats , F. Aieta , J.‐P. Tetienne , F. Capasso , Z. Gaburro , Science 2011, 334, 333. 21885733 10.1126/science.1210713

[36] X. Pan , Y. Deng , Z. Cai , Z. Chen , Y. Ding , Z. Zheng , F. Ding , Adv. Sci. 2025, 12, 2413138. 10.1002/advs.202413138 PMC12061329 39965134

[37] M. Liu , Q. Yang , A. A. Rifat , V. Raj , A. Komar , J. Han , M. Rahmani , H. T. Hattori , D. Neshev , D. A. Powell , I. V. Shadrivov , Adv. Opt. Mater. 2019, 7, 1900736.

[38] X. Y. Wu , H. Y. Feng , F. Wan , M. Wei , C. Guo , L. Cai , F. Wu , Z. H. Jiang , L. Kang , W. Hong , D. H. Werner , Adv. Mater. 2024, 36, 2402170. 10.1002/adma.202402170 38587064

[39] Z. Yue , J. Li , J. Liu , J. Li , C. Zheng , G. Wang , H. Xu , M. Chen , Y. Zhang , Y. Zhang , J. Yao , Adv. Opt. Mater. 2022, 10, 2200733.

[40] P. Yu , J. Li , N. Liu , Nano Lett. 2021, 21, 6690. 34286586 10.1021/acs.nanolett.1c02318 PMC8361430

[41] P. Fei , G. A. E. Vandenbosch , W. Guo , X. Wen , D. Xiong , W. Hu , Q. Zheng , X. Chen , Adv. Opt. Mater. 2020, 8, 2000194.

[42] S. Teng , Q. Zhang , H. Wang , L. Liu , H. Lv , Photonics Res. 2019, 7, 246.

[43] H. Jeong , D. H. Le , D. Lim , R. Phon , S. Lim , Adv. Opt. Mater. 2020, 8, 1902182.

[44] F. Costa , A. Monorchio , G. Manara , Appl. Comput. Electromagnet. Soc. J. 2014, 29, 960.

[45] W. Liu , H. Cheng , J. Tian , S. Chen , Adv. Phys.: X 2020, 5, 1742584.

[46] B. Li , W. Piyawattanametha , Z. Qiu , Micromachines 2019, 10, 310. 31071944 10.3390/mi10050310 PMC6562435

[47] Z. Xing , Z. Lin , N. Liu , H. Gao , Y. Hu , Z. Liu , Z. Jiang , X. Zhang , C. Zhang , Laser Photonics Rev. 2025, 19, 2401993.

[48] H. Ahmed , H. Kim , Y. Zhang , Y. Intaravanne , J. Jang , J. Rho , S. Chen , X. Chen , Nanophotonics 2022, 11, 941. 39634470 10.1515/nanoph-2021-0746 PMC11501437

[49] P.‐Y. Chen , J. Soric , A. Alù , Adv. Mater. 2012, 24, OP281. 23080411 10.1002/adma.201202624

[50] W. Wan , J. Gao , X. Yang , Adv. Opt. Mater. 2017, 5, 1700541.

[51] A. Isichenko , N. Chauhan , D. Bose , J. Wang , P. D. Kunz , D. J. Blumenthal , Nat. Commun. 2023, 14, 3080. 37248247 10.1038/s41467-023-38818-6 PMC10227028

[52] C. Ropp , W. Zhu , A. Yulaev , D. Westly , G. Simelgor , A. Rakholia , W. Lunden , D. Sheredy , M. M. Boyd , S. Papp , A. Agrawal , Light: Sci. Appl. 2023, 12, 83. 37009814 10.1038/s41377-023-01081-x PMC10068800

[53] W. R. McGehee , W. Zhu , D. S. Barker , D. Westly , A. Yulaev , N. Klimov , A. Agrawal , S. Eckel , V. Aksyuk , J. J. McClelland , New J. Phys. 2021, 23, 013021.

[54] L. Zhu , X. Liu , B. Sain , M. Wang , C. Schlickriede , Y. Tang , J. Deng , K. Li , J. Yang , M. Holynski , S. Zhang , T. Zentgraf , K. Bongs , Y.‐H. Lien , G. Li , Sci. Adv. 2020, 6, abb6667. 10.1126/sciadv.abb6667 PMC7439576 32832692

[55] M. Jin , X. Zhang , X. Liu , C. Liang , J. Liu , Z. Hu , K. Li , G. Wang , J. Yang , L. Zhu , G. Li , Nano Lett. 2023, 23, 4008. 37098832 10.1021/acs.nanolett.3c00791

[56] L. Wu , J. Tao , G. Zheng , Phys. Rev. B 2018, 97, 245426.

[57] T. Tian , Y. Liao , X. Feng , K. Cui , F. Liu , W. Zhang , Y. Huang , Adv. Opt. Mater. 2023, 11, 2300664.

[58] Z. Ma , T. Tian , Y. Liao , X. Feng , Y. Li , K. Cui , F. Liu , H. Sun , W. Zhang , Y. Huang , Nat. Commun. 2024, 15, 8370. 39333169 10.1038/s41467-024-52676-w PMC11436973

[59] F. Ding , B. Chang , Q. Wei , L. Huang , X. Guan , S. I. Bozhevolnyi , Laser Photonics Rev. 2020, 14, 2000116.

[60] Y. Deng , C. Wu , C. Meng , S. I. Bozhevolnyi , F. Ding , ACS Nano 2021, 15, 18532. 34779618 10.1021/acsnano.1c08597

[61] S. im Sande , Y. Deng , S. I. Bozhevolnyi , F. Ding , Opto‐Electron. Adv. 2024, 7, 240076.

