# Supplementary material for: Polarization Decoupling Multi‐Port Beam‐Splitting Metasurface for Miniaturized Magneto‐Optical Trap
Source: Adv Sci (Weinh). 2025 Jul 12;12(37):e06289. doi: 10.1002/advs.202506289 (PMC12499503; doi:10.1002/advs.202506289)
Supplement: Supplementary file 1 — Supporting Information [file ADVS-12-e06289-s001.docx]

Supporting Information for:

**Polarization Decoupling Multi-Port Beam-Splitting Metasurface for Miniaturized Magneto-Optical Trap**

Tian Tian^1^, Chen Qing^2^, Yuxuan Liao^1^, Jiajun Zhu^2^, Yongzhuo Li^1^, Xue Feng^1^*, Dengke Zhang^2^*and Yidong Huang^1^*

^1^Department of Electronic Engineering, Tsinghua University, Beijing 100084, China

^2^School of Instrumentation and Optoelectronic Engineering, Beihang University, Beijing 100191, China

*Corresponding author: [x-feng@tsinghua.edu.cn](mailto:x-feng@tsinghua.edu.cn); [dkzhang@buaa.edu.cn](mailto:dkzhang@buaa.edu.cn); [yidonghuang@tsinghua.edu.cn](mailto:yidonghuang@tsinghua.edu.cn)

**S1. The customized hexagonal prism glass chamber**

The schematic diagram of the custom-designed hexagonal prism chamber is presented in Figure S1a. The chamber is constructed with transparent quartz glass to facilitate the transmission of cooling laser beams. Inside the chamber, rubidium (Rb) atomic vapor is filled. The height of the hexagonal prism is 39 mm, while a face-to-face distance is 27 mm across the hexagonal cross-section. Besides, the sidewall thickness of the chamber is 2 mm. All metasurfaces are adhered on its outside surfaces. For more clarity, Figure S1b provides a detailed illustration of the optical path. As depicted, one of the splitting sub-beams deflecting from the PD-MPBS metasurface (MS-BS) propagates approximately 16.7 mm before impinging on reflective beam-expanding metasurface (MS-RE). Subsequently, the sub-beam is reflected and converges with other sub-beams at the center inside the chamber after propagating an additional distance of 16.7 mm.

**
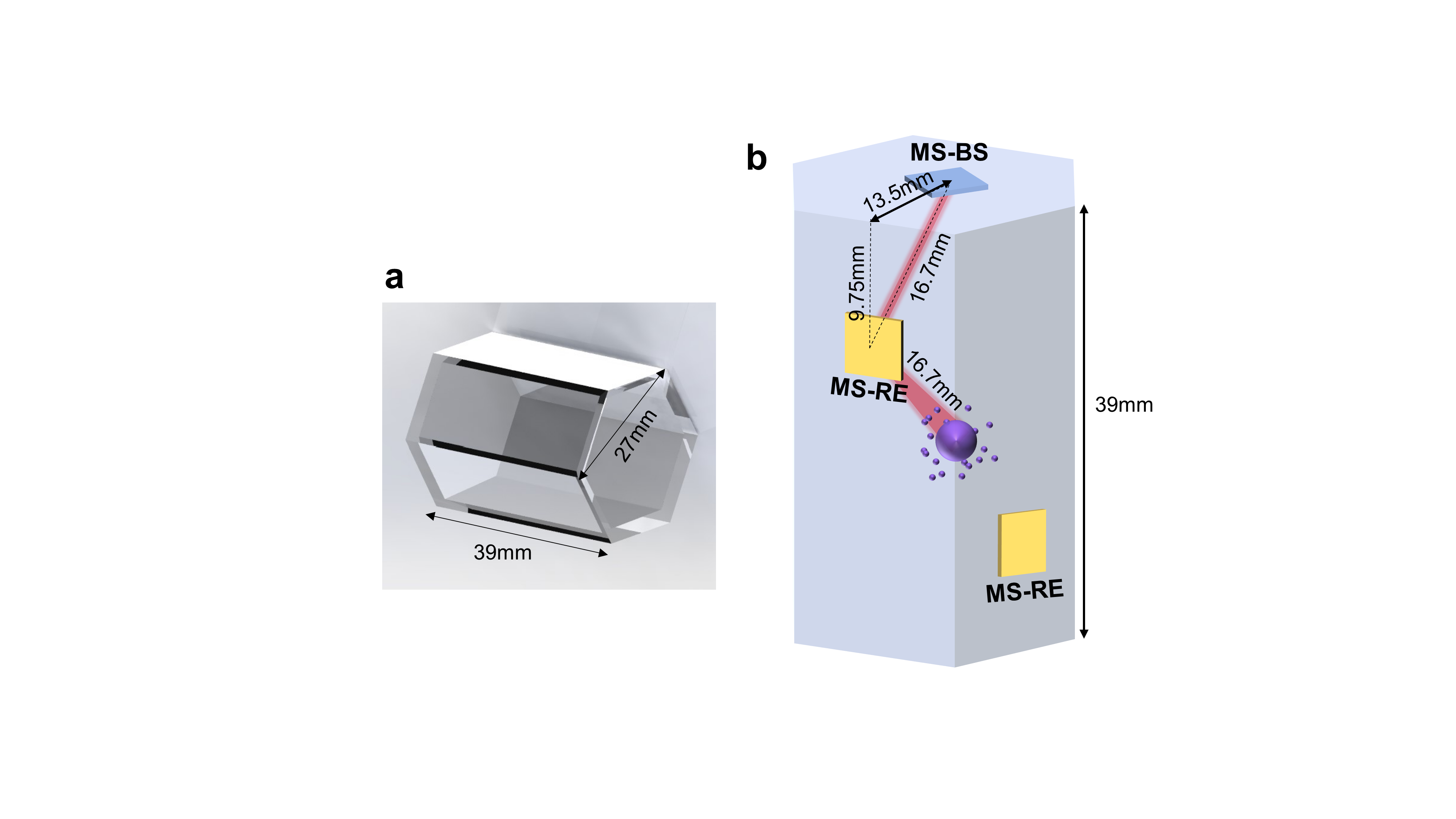
**

**Figure S1.** The schematic of the customized hexagonal prism glass chamber. (a) The 3D schematic of the hexagonal prism chamber. (b) The detailed illustration of the optical path.

**S2. The optimization results of two-port beam-splitting phase pattern**

For the output LCP component of the PD-MPBS metasurface, the target phase function (denoted as $\phi_{2}$) is designed to achieve two-port beam splitting with both deflection angles of 54.7°. A straightforward approach is to employ the superposed blazed grating. The visualized phase pattern of the superposition of two blazed gratings is shown in Figure S2a. The corresponding output optical field is calculated by discrete Fourier transform (DFT)-based light field simulation method and presented in Figure S2b. It can be seen that there is stray light in the output filed, primarily attributed to the limitation of phase-only modulation, which is unable to precisely accomplish simultaneous amplitude and phase modulation. To address this issue, a gradient-based iterative optimization algorithm is utilized to derive the optimal phase-only modulation pattern, which generates a light field distribution on the observation plane that closely approximates the desired ideal distribution. The basic principles and procedure can be acquired in our previous work^[1]^. The optimized phase pattern of $\phi_{2}$ is presented in Figure S2c, which contains more meticulous details compared to the original one. Besides, the simulated output field corresponding to the optimized phase pattern is shown in Figure S2d, revealing the effective suppression of undesired light and yielding a more precise target light field distribution.

**
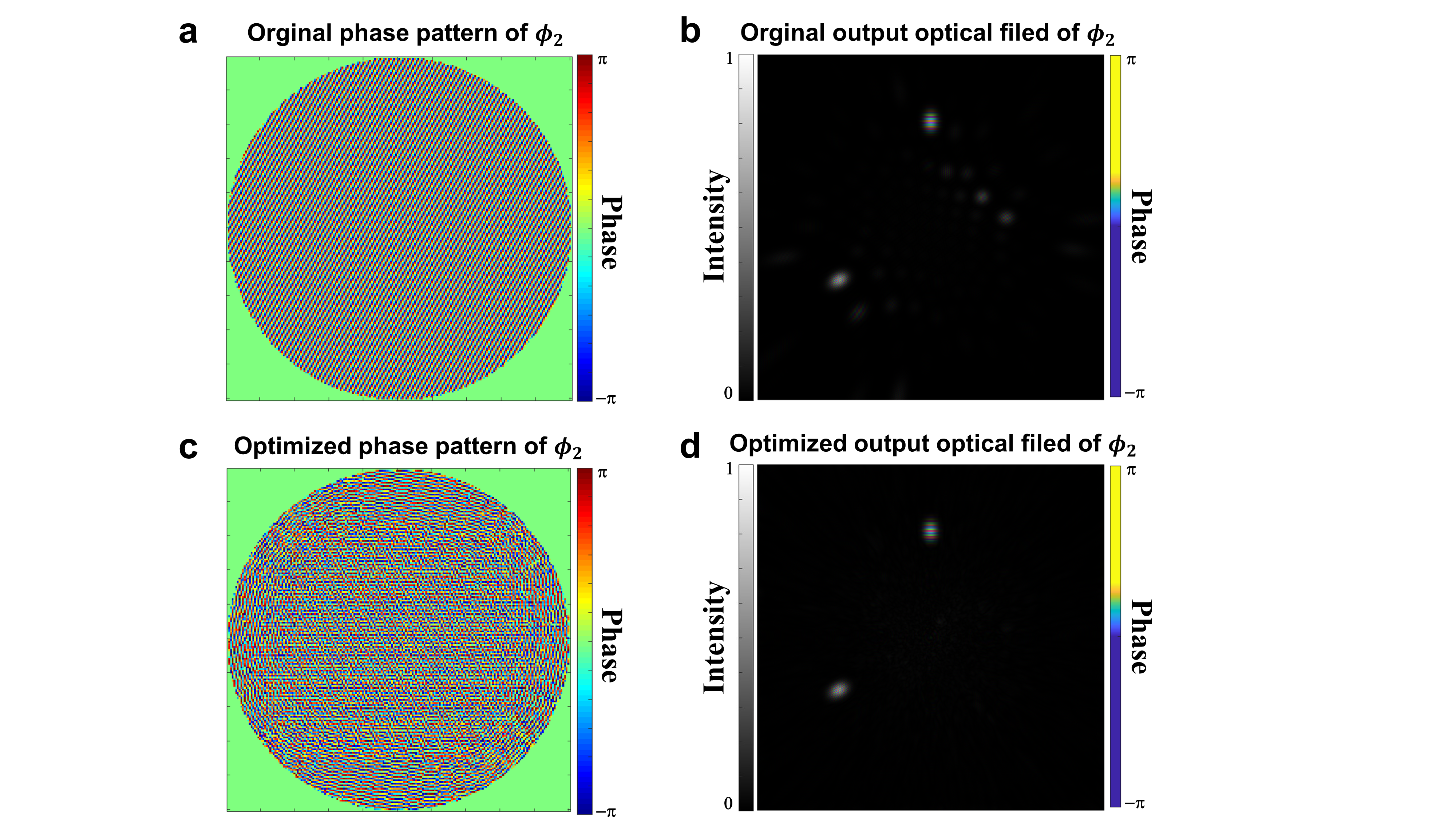
**

**
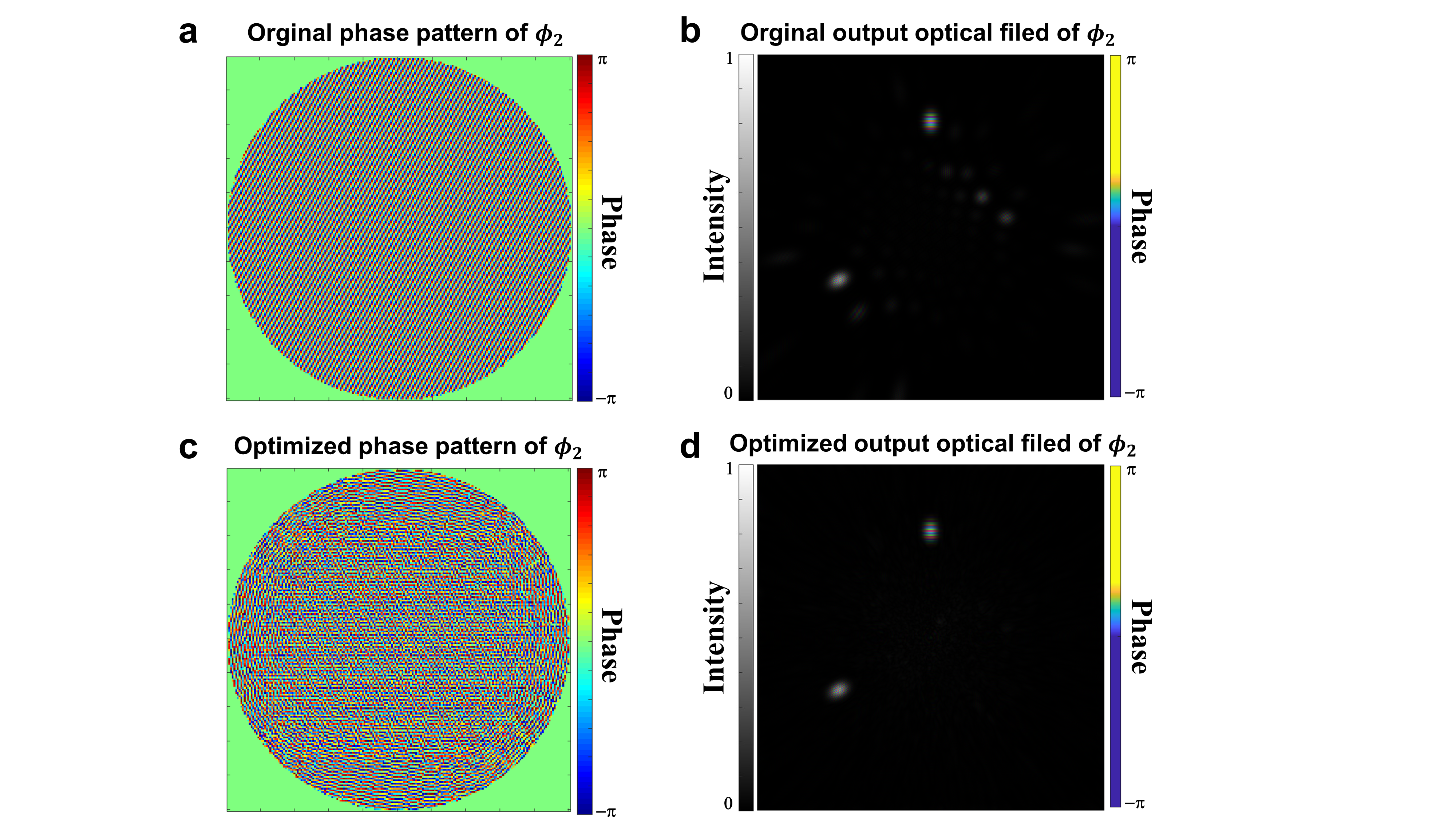
**

**Figure S2.** The optimization results of two-port beam-splitting phase pattern. (a) The original phase pattern of $\phi_{2}$ obtained by the superposition of two blazed gratings. (b) The output optical field of the original phase pattern. (c) The optimized phase pattern of $\phi_{2}$ obtained by gradient-descent-based algorithm. (d) The output optical field of the optimized phase pattern.

**S3.** **Experimental measurement for the dielectric function of α-Si sample**

To obtain accurate design, we have measured the dielectric function of amorphous silicon (α-Si) deposited on a quartz substrate. Initially, a 400 nm thick layer of quartz was sputter-deposited on a commercial crystalline silicon (Si) substrate, followed by the deposition of a 500 nm thick layer of α-Si with plasma-enhanced chemical vapor deposition (PECVD). It was designed to mimic the α-Si metasurface structure on a quartz substrate. The Si substrate was employed to reduce the transparency of the sample, thereby improving the accuracy of ellipsometry measurements. After sample preparation, the refractive index (*n*) and absorption coefficient (*k*) of α-Si material were measured within the wavelength range of 450 nm to 1550 nm by a spectroscopic ellipsometer (Eoptics ME-L). The results shown Figure S3 indicate that at the design wavelength of 780 nm, the refractive index of the α-Si is approximately *n*=3.64, and the absorption coefficient is about *k*=0.028.


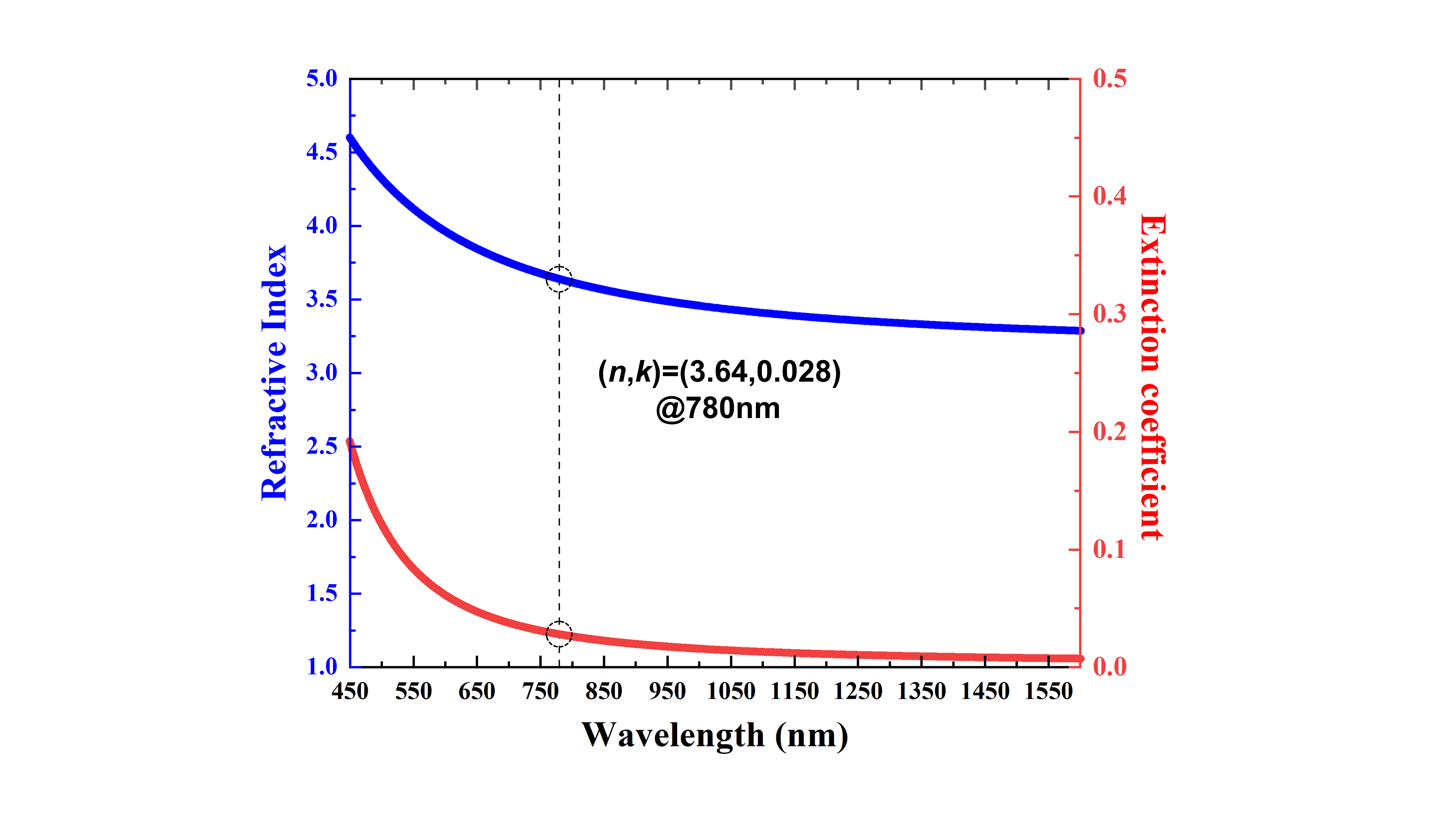


**Figure S3.** The experimentally measured dielectric function of the α-Si sample.

**S4. The analysis of broadband characteristics of the designed PD-MPBS metasurface**

To evaluate the broadband characteristics of the designed PD-MPBS metasurface, we have performed the simulation analysis of the geometries and corresponding transmittance/phases as a function of the wavelength. As depicted in Figure S4, for the designed 143 meta-atoms, we simulated the phase retardation ($\phi_{RR}$), the transmittance ($A_{RR}$ for co-polarization and $A_{RL}$ for cross-polarization components) and polarization conversion efficiency (*PCE*) across an 80nm bandwidth (740 nm~820 nm) near the designed wavelength. Among them, the black data points represent the results at the designed wavelength of 780 nm. As can be observed, the designed 143 meta-atoms can cover the full 2π phase retardation range while maintaining $PCE\approx2/3$ (i.e., $A_{RR}\approx\sqrt{1/3}$ and $A_{RL}\approx\sqrt{2/3}$). By comparison, when the operating wavelength deviates from the designed one, the phase retardation also shifts from the designed value, but the overall trend of variation is preserved, and the 2π phase coverage is still maintained (Figure S4a). Since the metasurface is constructed based on relative phase gradients, this implies that, even at off-design wavelengths, these meta-atoms can still achieve the mapping of the target phase template. On the other hand, the transmittance ($A_{RR}$ and $A_{RL}$) and *PCE* exhibit relatively significant deviation from the designed wavelength condition. This indicates that the splitting ratio between the two orthogonal polarization components no longer maintains the designed 1:2 proportion, which may lead to non-uniform intensities among the trapping beams and consequently degrade the performance of the MOT.

To summarize, for an 80 nm bandwidth, the PD-MPBS metasurface could still achieve beam-splitting functionality, albeit with distortion in the power ratio. Considering the stringent requirement of laser beam uniformity in MOT, the proposed metasurface is recommended for single-wavelength operation.


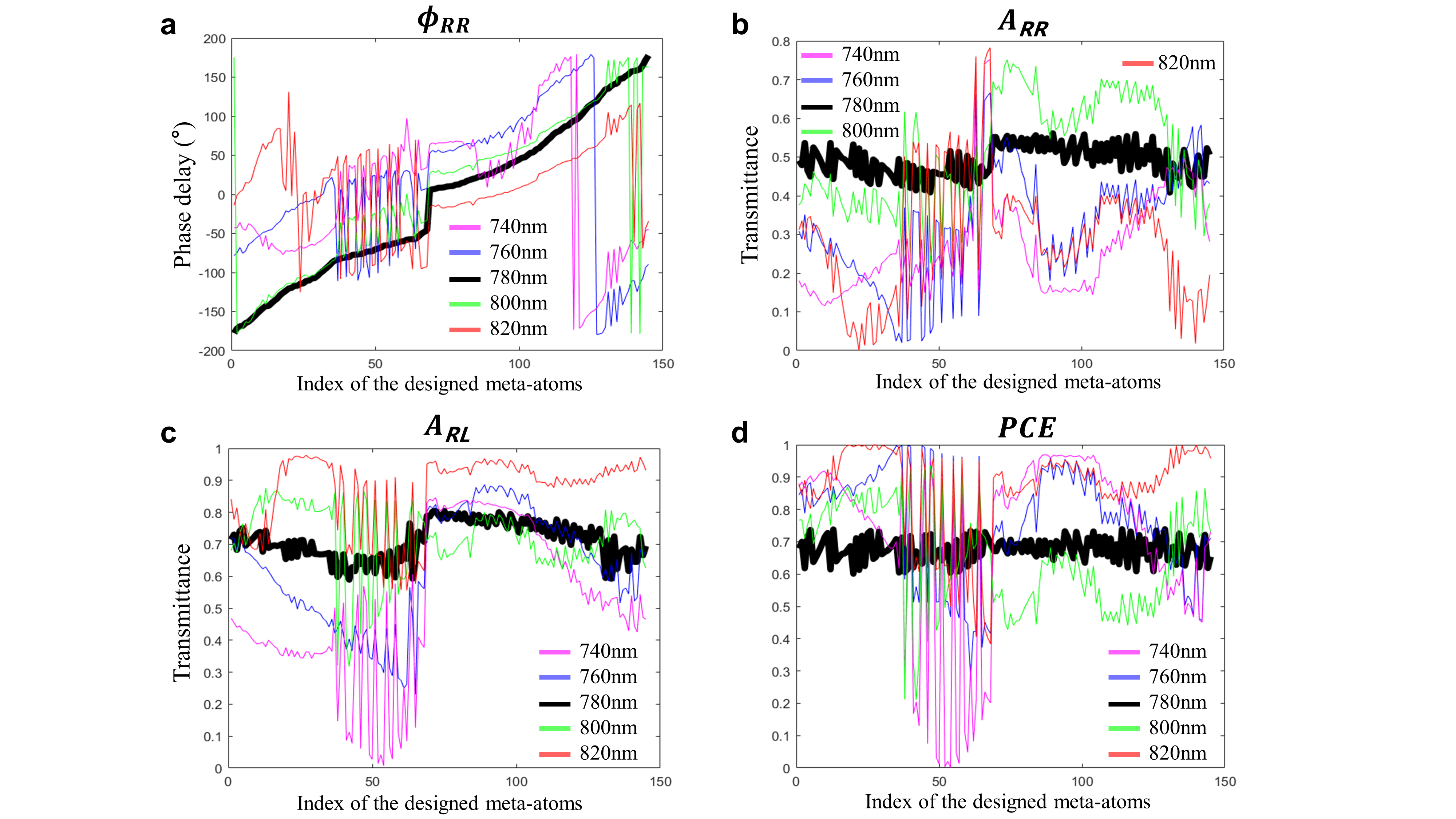


**Figure S4.** The broadband analysis of the designed PD-MPBS metasurface. (a-d) The simulated phase retardation $\phi_{RR}$ (a); and transmittance ($A_{RR}$ for co-polarization component (b); $A_{RL}$ for cross-polarization component (c)) and *PCE* (d) within 80 nm bandwidth near the designed wavelength.

**S5.** **The fabrication process of the metasurface samples**

**S5.1 The fabrication process of the PD-MPBS metasurface**

The detailed preparation process of the PD-MPBS metasurface is illustrated in Figure S5. Firstly, a 500 nm thick α-Si layer was deposited on a 500 μm thick quartz substrate via PECVD. Subsequently, a 100 nm thick chromium (Cr) layer was deposited on the sample through electron beam evaporation (EBE) to serve as a metal hard mask for the subsequent etching process. A layer of negative electron beam photoresist (ZEON ZEP520A) was then spin-coated on the sample and exposed with electron beam lithography (EBL). The pattern from the electron beam resist layer was transferred to the Cr mask layer by inductively coupled plasma (ICP) etching of the Cr layer. The α-Si layer was then etched using inductively coupled plasma reactive ion etching (ICP-RIE), and the Cr mask was removed by the chromium etchant (a mixture of nitric acid, phosphoric acid and hydrofluoric acid). Ultimately, the designed metasurface pattern was obtained on the α-Si layer.


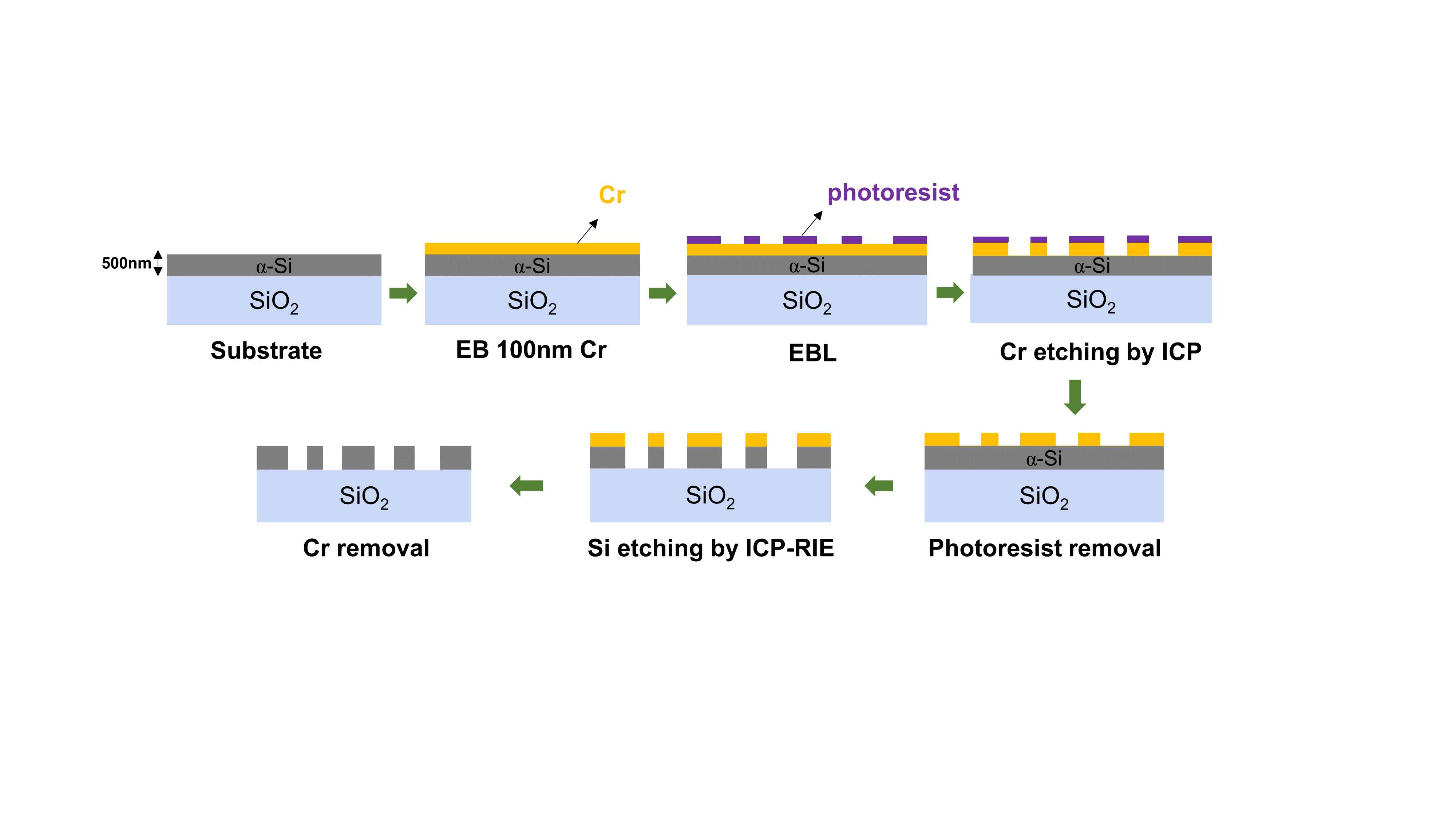


**Figure S5.** The fabrication process of the PD-MPBS metasurface.

**S5.2 The fabrication process of the reflective beam-expanding metasurface**

As mentioned in the main text, during the fabrication of the reflective beam-expanding metasurface, a 5 nm Cr adhesion layer was incorporated between each gold (Au) layer and the dielectric layer to prevent the Au layer from peeling off due to poor adhesion. The actual structure of the sample is illustrated in Figure S6a. The detailed fabrication process is described in Figure S6b: Firstly, a 5 nm Cr layer was deposited on a crystalline silicon (Si) substrate as an adhesion layer by EBE. Next, a 100 nm Au layer was deposited via EBE to serve as the bottom reflective layer of the metasurface, followed by another 5 nm Cr layer deposited as an adhesion layer. Subsequently, a 60 nm thick silicon dioxide (SiO_2_) layer was deposited via magnetron sputtering process. And then, the negative electron beam resist (ZEON ZEP520A) was spin-coated on the multilayer film and evenly distributed, followed by exposure using EBL to define the metasurface pattern. Then, a 20 nm thick Au layer was deposited by EBE. Finally, the electron beam photoresist was removed through lift-off process, transferring the metasurface pattern to the top Au layer and forming an array of Au rectangular nanofins.


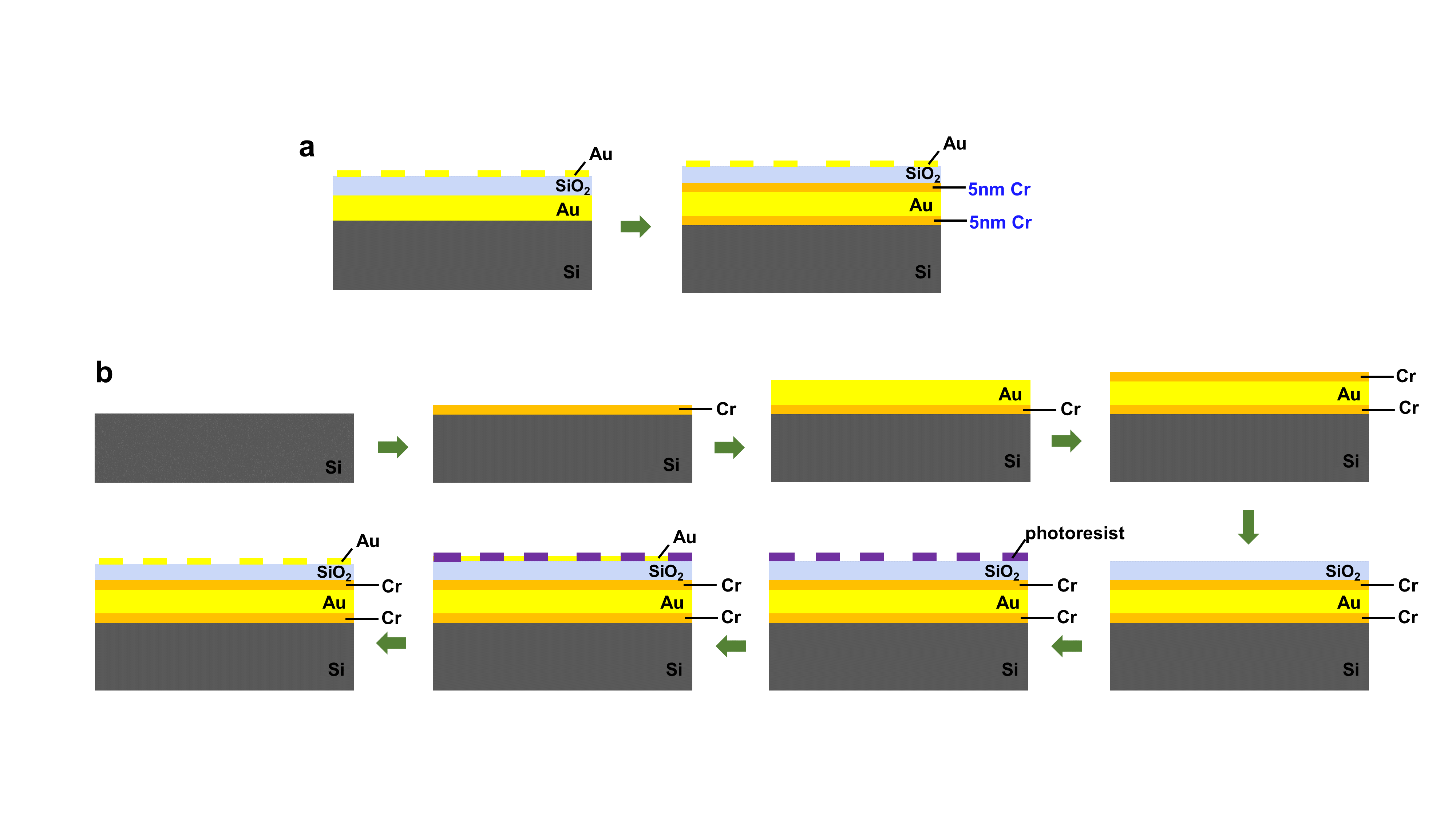


**Figure S6.** The fabrication process of the MIM-type reflective beam-expanding metasurface. (a) The designed and practically fabricated structure of the reflective beam-expanding metasurface. (b) The fabrication process of the reflective beam-expanding metasurface.

**S6. The complete characterization results of two fabricated PD-MPBS samples**

Table S1 summarizes the complete characterization results of two fabricated PD-MPBS samples, including the measured power, splitting angles and circular polarization purities of the sub-beams. In the experiment, the incident power on both samples is 27 mW. For sample1, the total power of three sub-beams is about 6.6 mW, indicating the modulated efficiency of 24.2%. Besides, the power differences between the three sub-beams are within 4.4%, which reveals high beam-splitting ratio fidelity for the proposed PD-MPBS meta-device. Additionally, the average splitting angle is measured as 54.5° and the polarization purities exceed 91.3%. For sample2, similar characterization results are also verified. The modulated efficiency is about 24.1% along with power difference less than 3.2%, and the average splitting angle and polarization purity are 54.5° and 92.6%, respectively.

**Table S1.** The complete characterization results of two fabricated PD-MPBS samples

|  | **Power(mW)** | **Splitting angle** | **Polarization Purity** |
| --- | --- | --- | --- |
| **Sample1 Sub-beam 1** | 2.26 | 54.6° | 93.2%(RCP) |
| **Sample1 Sub-beam 2** | 2.16 | 54.4° | 91.3%(LCP) |
| **Sample1 Sub-beam 3** | 2.18 | 54.5° | 92.8%(LCP) |
| **Sample2 Sub-beam 1** | 2.20 | 54.4° | 92.8%(RCP) |
| **Sample2 Sub-beam 2** | 2.13 | 54.7° | 92.0%(LCP) |
| **Sample2 Sub-beam 3** | 2.18 | 54.5° | 93.1%(LCP) |

**S7. The analysis of the impact of fabrication errors on the performances of PD-MPBS metasurface**

By analyzing the discrepancies between the characterization images of the fabricated samples (Figure 3 in the main text) and the design layout, we identify two primary fabrication errors causing deviations in the fabricated rectangular pillars from their design specifications. One is the dimensional deviation, where the length and width of the fabricated nanofins differ from the design values, mainly attributed to the finite precision of EBL. For our fabrication process, the dimensional deviation can be controlled within 10 nm range (i.e., *ΔL* and *ΔW* < 10 nm). The second is the rough sidewalls of the rectangular nanofins, inevitably introduced by the etching process.

To simulate the impact of these two fabrication errors on the performance of the fabricated device, through FDTD simulation, we incorporated both types of errors for each rectangular α-Si nanofin (as illustrated in Figure S7c). Specifically, the length and width deviations (*ΔL* and *ΔW*) with Gaussian distributions (mean value:0; standard deviation: 3.3 nm) are added to each meta-atom, and a simulated fluctuation with Gaussian distribution (mean value: 0; standard deviation: 1 nm) is also added to the geometric edge of each meta-atom to mimic the sidewall roughness. For the 3-port PD-MPBS metasurface, Figures S7b and S7d show the comparison of the output optical fields for the ideal and fluctuated case. It reveals a significant increase in the proportion of the 0-order central spot (unmodulated direct transmission), indicating a reduction in the diffraction efficiency of the device. For the fluctuated case, the central spot accounts for approximately 29.4%, which is consistent with the experimentally observed results.


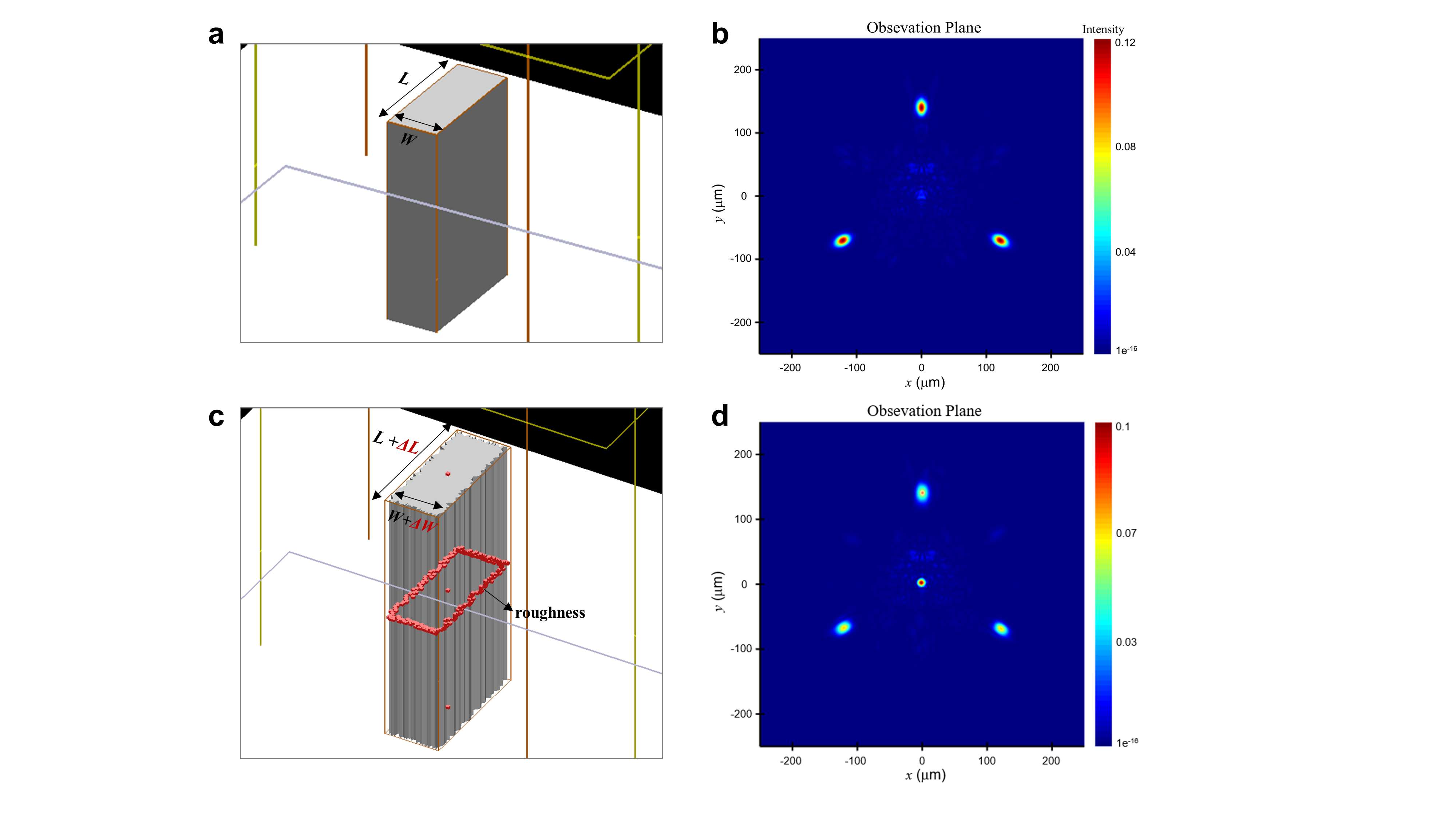


**Figure S7.** Simulation analysis of how the two aspects of fabrication errors affect the PD-MPBS metasurface performance. (a,b) The designed nanofin and the corresponding output optical field for the ideal metasurface. (c,d) The nanofin with two fabrication errors and the corresponding output optical field for the fluctuated metasurface.

Besides the diffraction efficiency, we also quantified the impact of fabrication errors on polarization purity and beam splitting ratio. Based on the results in Figure S7d, the polarization purity of each splitting sub-beam was calculated and summarized in Table S2. It can be seen that the fabrication errors cause a polarization purity degradation of approximately 5% for an individual sub-beam. Furthermore, while the beam-splitting ratio in the ideal simulation is nearly perfect (1:1:1), the splitting ratio incorporating fabrication errors becomes approximately 1:0.96:0.95, which is similar with the experimental results (Table S1).

Based on the above analysis, dimensional deviations and sidewall roughness are identified as the primary factors responsible for the discrepancy between the experimental and simulated performance of the PD-MPBS metasurface. With improved fabrication process, the diffraction efficiency, polarization purity and beam-splitting fidelity are expected to be further enhanced.

**Table S2.** The simulated polarization purities without and with consideration of fabrication errors

|  | **Sub-beam 1** | **Sub-beam 2** | **Sub-beam 3** |
| --- | --- | --- | --- |
| **Ideal (without fabrication error)** | 99.1% (RCP) | 98.7% (LCP) | 98.5% (LCP) |
| **Fluctuated (with fabrication error)** | 94.4% (RCP) | 93.9% (LCP) | 93.2% (LCP) |

**S8. The simulated and experimental diffraction efficiency of the PD-MPBS metasurface**

In simulation (as shown in Figure S8), our constructed PD-MPBS metasurface achieves diffraction efficiency of approximately 45.3%, where the 0th-order spot nearly negligible under ideal conditions. This theoretical diffraction efficiency is lower than that of many transmissive metasurfaces (some of them can exceed 90%^[2-5]^). This reduced efficiency primarily stems from the large beam-splitting angle (54.7°) of our device, which fundamentally differs from small-angle metasurfaces operating under the paraxial approximation^[5]^. Such a large-angle beam splitting requires a high phase gradient, which leads to increased scattering loss and reduced mode coupling efficiency. Specifically, an extremely high phase gradient can induce phase jumps exceeding π/2 between adjacent unit cells. This abrupt phase change disrupts the local phase approximation, exciting non-propagating modes (e.g., surface waves) or causing random scattering, thereby preventing efficient energy concentration into the target diffraction order. Furthermore, the large gradient necessitates significant variations in meta-atom dimensions, resulting in resonant mode mismatch and degraded energy transfer efficiency^[6]^.


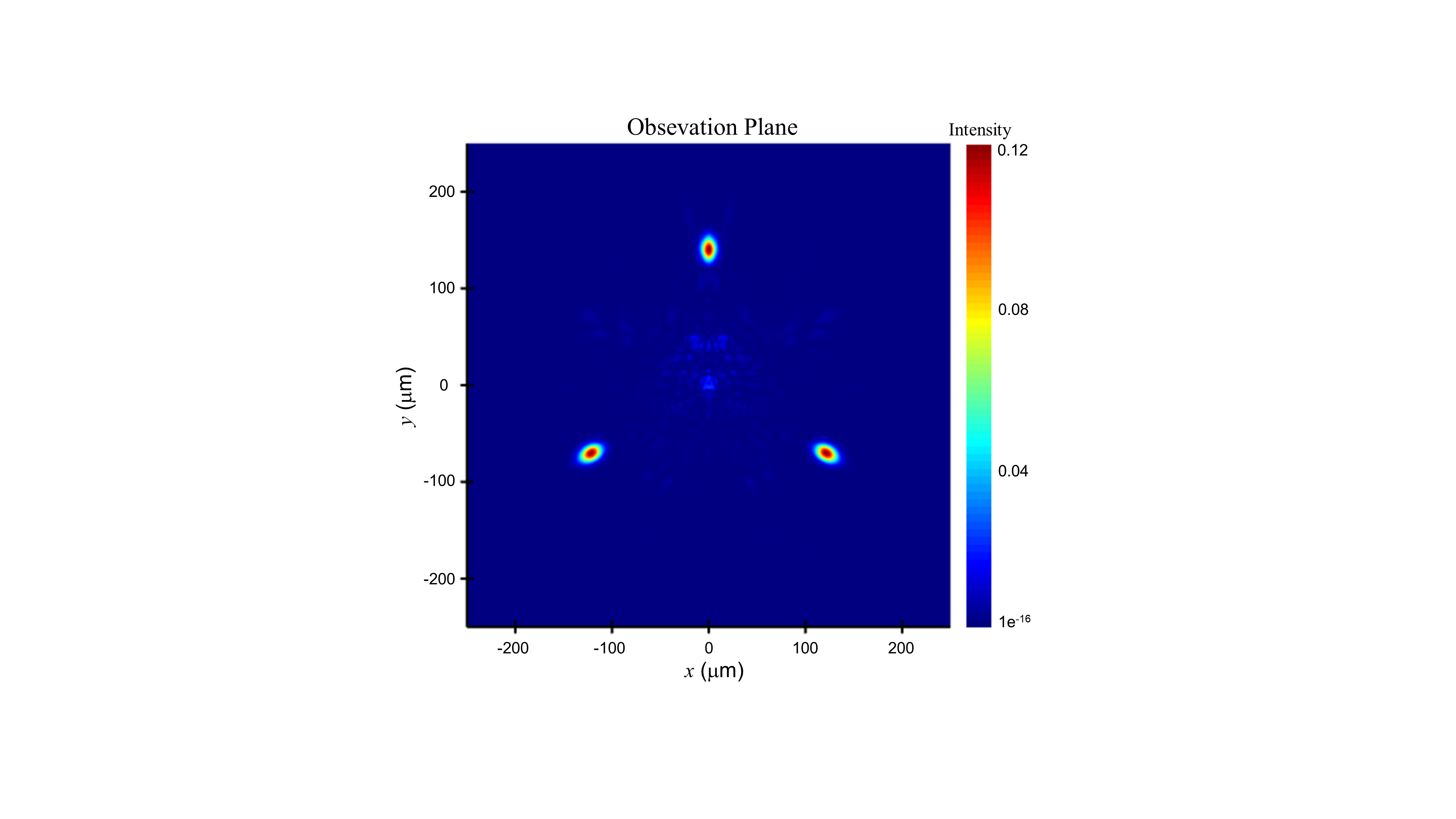


**Figure S8.** The simulated beam-splitting performance of the constructed PD-MPBS metasurface

In our experiments, the measured diffraction efficiency is 24.2% (complete experimental results can be found in Supporting Information S6), which is lower than the simulated value due to the presence of the 0th-order spot. Further improvement in experimental efficiency can be achieved by optimizing the fabrication process to reduce the proportion of the 0th-order spot, as detailedly discussed in Supporting Information S7.

.

**S9. The experimental results of beam delivery system setup for miniaturized MOT**

Figure S9 illustrates the beam expansion and overlapping characteristics under different scenarios: For comparison, Figure S9a presents the unmodulated 0th-order central spot of the PD-MPBS metasurface, exhibiting no observable beam expansion. Figure S9b exhibits the beam expansion effect of a single sub-beam upon reflection by the reflective beam-expanding metasurface, indicating a substantial increase in beam diameter. Figure S9c captures the spatial overlap of two sub-beams subsequent to their expansion by the reflective beam-expanding metasurfaces. Figure S9d portrays the overlapping pattern of three expanded beams, unveiling a "quasi-hexagonal star" distribution within the overlapping region. As elucidated in the main text, this phenomenon is ascribed to the 54.7° tilt angle between the detection plane and the beam propagation direction, resulting in axial stretching of each beam. Notably, when the detection plane is orthogonal to the beam propagation direction, the expanded beams preserve typical Gaussian mode field distribution, as illustrated in Figures S9b and S9c.


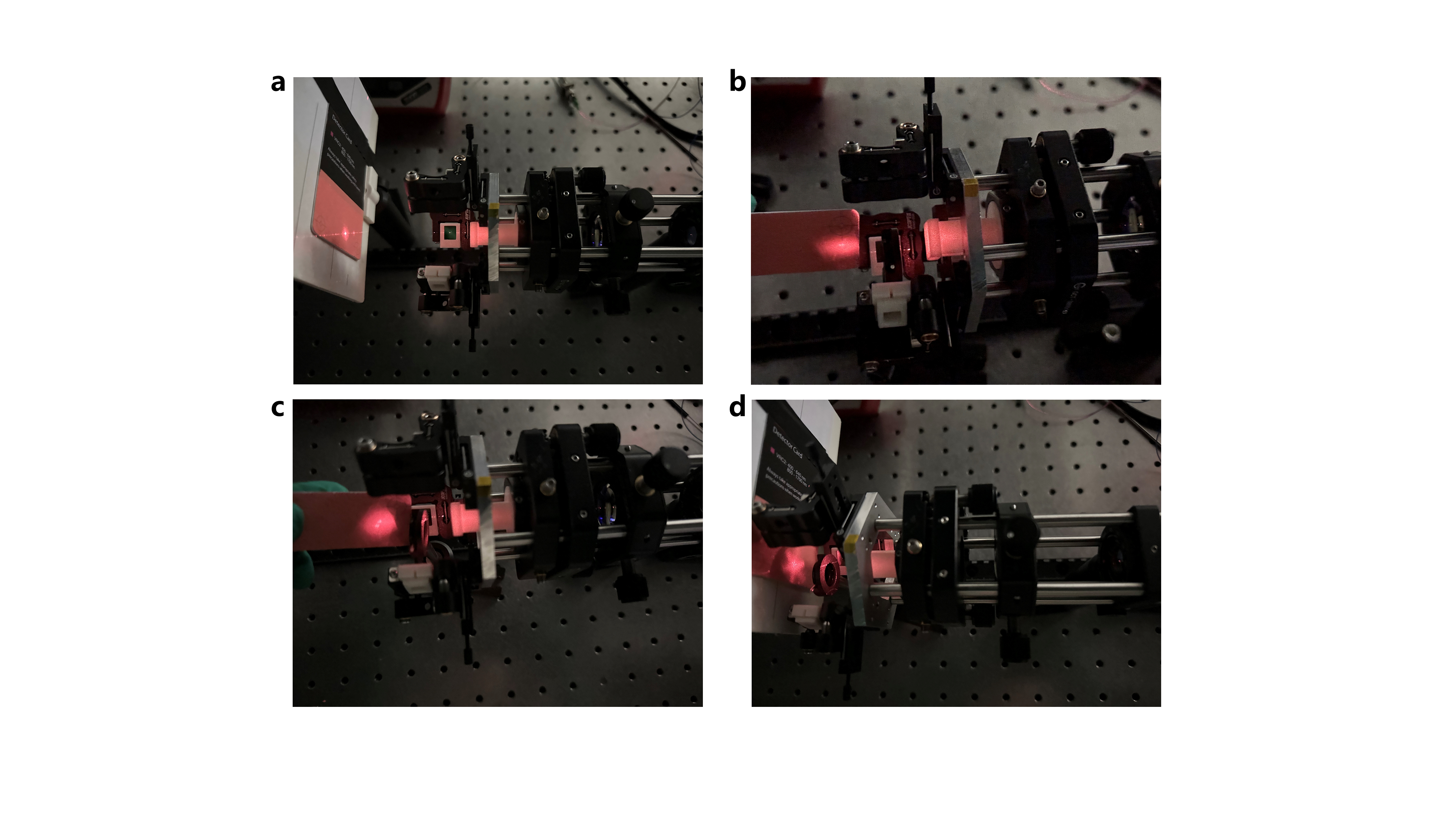


**Figure S9.** The supplementary experimental results of beam delivery system setup for miniaturized MOT. (a) The unmodulated central spot from the PD-MPBS metasurface (for comparison). (b) Beam expansion effect of a single sub-beam modulated by the reflective beam-expanding metasurface. (c) Spatial overlap of two sub-beams after expansion by the reflective beam-expanding metasurfaces. (d) Spatial overlap pattern of three expanded beams.

**S10. The assembly method for the metasurface-chamber integration and the influence of the chamber wall thickness**

At the preliminary experimental verification stage, we employed micro-adjustment mounts to hold and align the metasurfaces. However, after the customized gas chamber is prepared, the metasurface chip can be bonded to a silicon frame, forming a hybrid chip with the metasurface pattern surrounded by the silicon frame as shown in Figure S10a. During assembly, epoxy adhesive is uniformly applied to the peripheral silicon frame, enabling precise bonding between the metasurface-silicon hybrid chip and the chamber. With this approach, all metasurfaces can be attached to the exterior surfaces of the gas chamber, thus achieving mounts-free metasurface-chamber assembly. The optimal position of attachments can be readily determined through experimental characterization in advance. As a result, after the bounding and attachment, the system volume is predominantly determined by the gas chamber.

Figure 1c (in the main text) provides a conceptual schematic of our proposed metasurface-based beam delivery system. However, based on the assembly method of the metasurfaces and chamber mentioned above, the actual configuration is shown in Figure S10b. This assembly strategy provides three key benefits:

(1) This effectively prevents direct contact between the metasurface nanostructures and the chamber walls, thereby eliminating potential damage to the delicate nano-features while significantly simplifying the assembly process.

(2) This configuration ensures the operation of the metasurface according to its designed functionality. By maintaining physical separation from the vapor chamber walls, the metasurface preserves its original refractive index distribution environment, thus remaining completely immune to any optical influence from the chamber boundaries. To mitigate reflections at the chamber walls, we employ conventional anti-reflection coatings—a well-established solution widely used in MOT configurations. This approach effectively minimizes optical losses and completely eliminates the influence of the chamber walls on the optical performance of metasurface.

(3) The optical path can be optimized by tuning the thickness of the hollow silicon spacer. Due to the thickness of the vapor chamber (2mm), the transmitted light undergoes both reflection and refraction at the chamber walls. For the reflections, we employ standard anti-reflection coatings to effectively mitigate these reflective losses. For the refractions, Since the chamber walls consist of parallel glass plates, the angles of incident and outgoing light remain unchanged, but a slight displacement occurs. This displacement effect can be compensated by optimizing the silicon spacer thickness (the distance between the metasurface and chamber) and precisely adjusting the position of metasurface. For example, when the distance from the reflective metasurface to the chamber is fixed at $t_{\text{RE}}=0.5\text{mm}$ (with air refractive index *n* = 1 and glass refractive index *n* = 1.45), setting the spacer thickness of PD-MPBS $t_{\text{BS}} = 0.5 \text{mm}$ and properly adjusting the positions of reflective metasurfaces enables precise beam focusing at the center of the atomic vapor chamber. The corresponding beam propagation diagram is illustrated in Figure S10b.


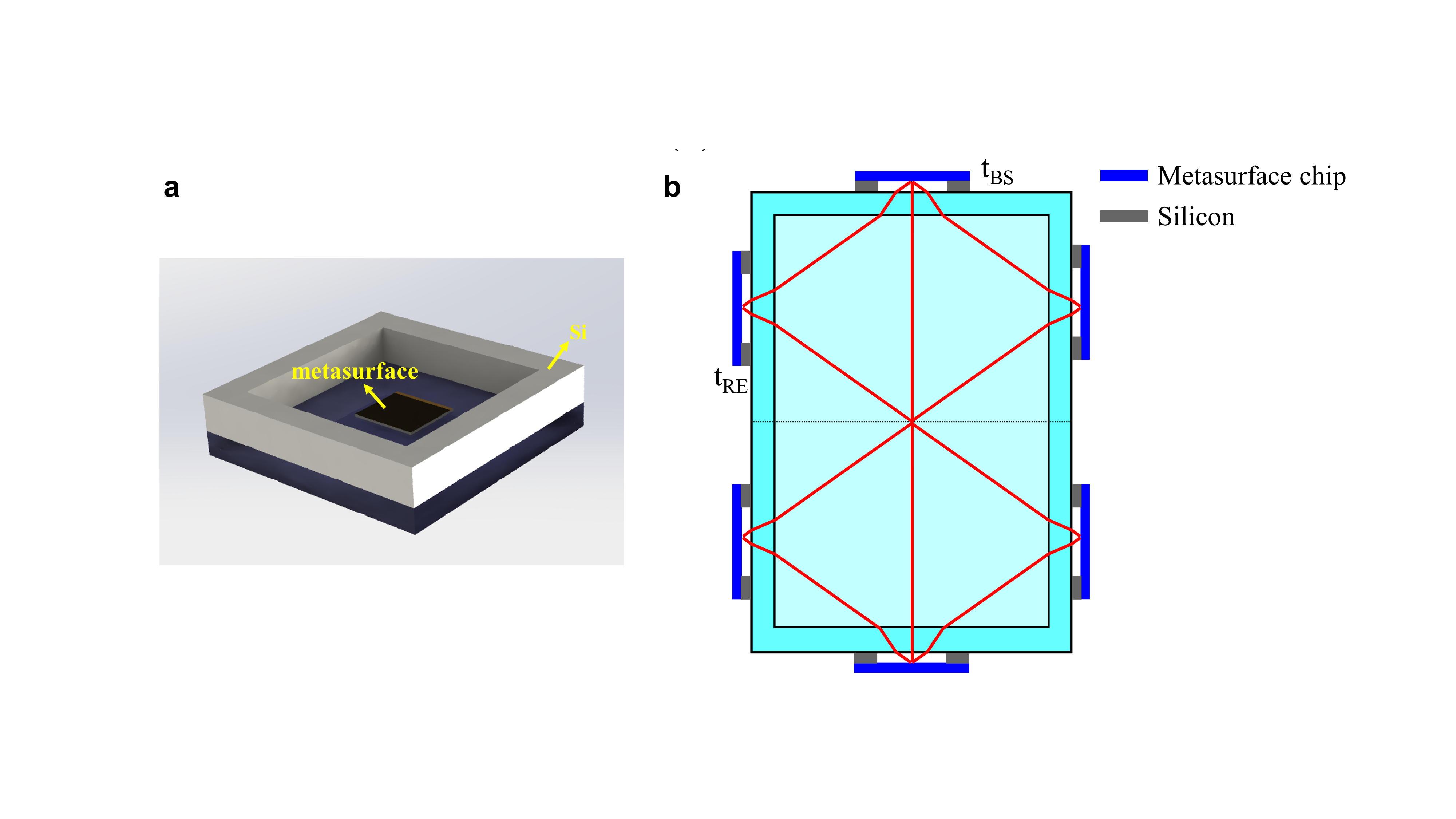


**Figure S10.** (a) The metasurface-chamber assembly method. (b) The actual experimental optical path diagram of the proposed metasurface-based MOT scheme.

**References:**

1. Tian, Tian, et al. "Metasurface‐based free‐space multi‐port beam splitter with arbitrary power ratio." *Advanced Optical Materials* 11.20 (2023): 2300664.
2. Shalaev, Mikhail I., et al. "High-efficiency all-dielectric metasurfaces for ultracompact beam manipulation in transmission mode." *Nano letters* 15.9 (2015): 6261-6266.
3. Abouelatta, Mahmoud AA, S. S. A. Obayya, and Mohamed Farhat O. Hameed. "Highly efficient transmissive metasurface for polarization control." *Optical and Quantum Electronics* 53 (2021): 1-11.
4. Chen, Wei Ting, et al. "Dispersion-engineered metasurfaces reaching broadband 90% relative diffraction efficiency." *Nature Communications* 14.1 (2023): 2544.
5. Chen, Xueyu, et al. "All-dielectric metasurface-based beam splitter with arbitrary splitting ratio." *Nanomaterials* 11.5 (2021): 1137.
6. Chu, Hongjun, et al. "Generalized rayleigh-sommerfeld diffraction theory for metasurface-modulating paraxial and non-paraxial near-field pattern estimation." *IEEE Access* 7 (2019): 57642-57650.
